# Comparative genomics of fatal ST308/O11 *Pseudomonas aeruginosa* from Egypt characterizes conserved resistance architecture, integron-associated multidrug resistance modules, and *blaNDM-1* resistance islands

**DOI:** 10.1186/s12866-026-05465-y

**Published:** 2026-08-08

**Authors:** Ahmed A. Sanad, Ghada A. El-Sherbeny, Adel A. El-Morsi, Ahmed F. Gad, Hossam I. Gebeer, Mohamed G. Seadawy

**Affiliations:** 1 Biodefense Center for Infectious and Emerging Diseases, Ministry of Defense, Cairo, 11775 Egypt; 2 Botany Department, Faculty of Science, Mansoura University, Mansoura, Egypt

**Keywords:** Ventilator-associated pneumonia, *Pseudomonas aeruginosa*, Pandrug-resistant, Whole-genome sequencing, Antimicrobial resistance, Virulence factors, ST308, Resistance nodulation division efflux systems

## Abstract

*Pseudomonas aeruginosa* is a major cause of ventilator-associated pneumonia (VAP) and is increasingly associated with extensive antimicrobial resistance, limiting therapeutic options and contributing to poor clinical outcomes. The emergence of high-risk clones that combine multidrug resistance and virulence-associated traits represents a growing public health concern.

In this study, 100 clinical *P. aeruginosa* isolates recovered from VAP patients were screened using culture-based identification and antimicrobial susceptibility testing. Two isolates exhibiting the most extensive resistance profiles, consistent with pandrug-resistant (PDR) phenotypes, and associated with fatal clinical outcomes were selected for whole-genome sequencing using Oxford Nanopore technology.

Comprehensive genomic analyses were performed to characterize antimicrobial resistance determinants, chromosomal resistance mutations, virulence-associated genes, mobile genetic elements, genomic islands, and phylogenomic relationships. Both isolates were assigned to sequence type ST308 and serogroup O11, a globally disseminated high-risk lineage. Core-genome phylogenomic analysis of 52 genomes identified 144,617 informative SNPs across 5,085 shared core genes and showed that the two Egyptian isolates differed by only 74 core-genome SNPs while clustering within the international ST308 lineage.

The isolates harbored an extensive resistome including *blaNDM-1*,* blaOXA-10*,* blaOXA-488*,* qnrVC1*,* rmtF*,* sul1*,* sul2*,* floR*, and *msrE*. High-confidence chromosomal resistance mutations were detected in *gyrA* (T83I), *parC* (S87L), *oprD* (Q402HfsTer99), *nalC*,* nalD*, and *pmrB*, supporting resistance to fluoroquinolones, carbapenems, and polymyxins. Two complete class 1 integrons and one In0 structure, all carrying the class 1 integrase IntI1, were identified, comprising integron-associated multidrug resistance modules containing *qnrVC1*,* aadA11*,* aac(6’)-II*,* dfrB5*,* sul1*,* qacEΔ1*,* rmtF*,* blaOXA-10*, and *blaPAC-1*. In contrast, *blaNDM-1* was located within a distinct ISVsa3-associated resistance island together with *ble* and *msrE*. Genomic island analysis further demonstrated the co-localization of key resistance determinants and the *exoU* virulence factor within predicted genomic islands.

Both isolates carried a highly conserved virulome, including *exoU*,* exoT*, type VI secretion system components, iron-acquisition systems, and biofilm-associated genes. Collectively, these findings indicate the convergence of resistance islands, integron-associated multidrug resistance modules, chromosomal resistance mechanisms, and virulence-associated determinants within a globally disseminated ST308/O11 high-risk clone associated with fatal VAP cases in Egypt.

To our knowledge, this study provides the first comprehensive genomic characterization of ST308/O11 *P. aeruginosa* exhibiting a pandrug-resistant phenotype based on the antimicrobial panel tested and associated with fatal ventilator-associated pneumonia in Egypt. Furthermore, by integrating cohort-level screening of 100 clinical isolates with comparative whole-genome analysis, this work provides additional insight into the genomic diversity of globally disseminated high-risk lineages through the accumulation of resistance and virulence determinants.

**Supplementary Information:**

The online version contains supplementary material available at 10.1186/s12866-026-05465-y.

## Introduction

Ventilator-associated pneumonia (VAP) remains one of the most serious healthcare-associated infections affecting critically ill patients receiving mechanical ventilation in intensive care units (ICUs). It is associated with prolonged hospitalization, increased healthcare costs, and substantial morbidity and mortality, particularly when caused by multidrug-resistant (MDR) Gram-negative pathogens. Despite advances in infection prevention and antimicrobial therapy, VAP continues to represent a major clinical challenge worldwide because of the increasing prevalence of antimicrobial resistance [1].

Among the bacterial pathogens responsible for VAP, *Pseudomonas aeruginosa* is particularly problematic because of its remarkable genomic plasticity, metabolic versatility, and capacity to acquire antimicrobial resistance through both chromosomal mutations and horizontal gene transfer [2]. The organism possesses multiple intrinsic and acquired resistance mechanisms, including β-lactamases, target-site mutations, efflux pump overexpression, porin alterations, and mobile genetic elements carrying antimicrobial resistance genes. These characteristics have contributed to the emergence of internationally disseminated high-risk clones that combine extensive antimicrobial resistance with enhanced persistence in hospital environments.

The World Health Organization (WHO) has classified carbapenem-resistant *P. aeruginosa* as a critical-priority bacterial pathogen requiring urgent research and surveillance efforts [3]. In parallel, the global increase of multidrug-resistant (MDR), extensively drug-resistant (XDR), and pandrug-resistant (PDR) isolates has substantially reduced therapeutic options and has been associated with poor clinical outcomes, particularly among critically ill patients [4–6]. In addition to antimicrobial resistance, successful clinical lineages frequently harbor virulence determinants involved in biofilm formation, iron acquisition, secretion systems, and host-cell interactions. The coexistence of resistance and virulence determinants within successful epidemic clones represents an increasing threat to patient management and hospital infection control [7–9].

Whole-genome sequencing (WGS) has become an essential approach for investigating the genetic architecture of clinically important bacterial pathogens. Beyond conventional resistome and virulome profiling, WGS enables comprehensive characterization of chromosomal resistance mutations, mobile genetic elements, class 1 integrons and integron-associated resistance regions, resistance islands, genomic islands, and phylogenomic relationships, thereby providing high-resolution insight into the genetic basis of antimicrobial resistance and bacterial adaptation., thereby providing high-resolution insight into the genetic basis of antimicrobial resistance and bacterial adaptation [10].

Among the internationally recognized high-risk clones, sequence type ST308 has emerged as an increasingly important lineage associated with hospital-acquired infections, carbapenem resistance, and multidrug resistance across multiple geographic regions. Recent studies have documented the widespread dissemination of ST308 in Europe, Asia, the Middle East, Africa, and South America, highlighting its successful adaptation to hospital environments and its frequent association with clinically important carbapenemase genes, including *blaNDM-1*,* blaVIM*, and *blaIMP* [11, 12]. For example, Benzaarate et al. reported the circulation of carbapenem-resistant and extensively drug-resistant ST308 isolates among high-risk clones in Morocco, whereas Rapoport et al. described the dissemination of NDM-1-producing ST308 isolates in Argentina, further supporting the international expansion of this lineage. In Egypt, recent genomic investigations have also reported the emergence of blaNDM-1-producing ST308 isolates, highlighting the increasing regional importance of this high-risk clone and emphasizing the need for expanded genomic surveillance [12]. Comparative genomic investigations have further demonstrated that ST308 possesses a highly adaptable accessory genome enriched with mobile genetic elements that facilitate the acquisition and persistence of antimicrobial resistance determinants while maintaining clinically important virulence-associated traits. Despite these advances, important aspects of the genomic diversity, resistance architecture, and global dissemination of ST308 remain incompletely characterized, particularly in underrepresented geographic regions where genome-based surveillance remains limited.

In Egypt, genome-scale investigations of high-risk *P. aeruginosa* clones remain scarce, and few studies have comprehensively characterized PDR isolates recovered from patients with VAP. Expanding regional genomic datasets is essential for improving the understanding of the local epidemiology of high-risk clones, identifying the genetic basis of antimicrobial resistance and virulence, and placing Egyptian isolates within the broader global genomic context.

In the present study, we performed an in-depth genomic characterization of the two *P. aeruginosa* isolates exhibiting the most extensive antimicrobial resistance profiles among a cohort of VAP-associated clinical isolates and associated with fatal clinical outcomes. Whole-genome sequencing and comparative genomic analyses were used to characterize antimicrobial resistance determinants, chromosomal resistance mutations, virulence-associated genes, integron-associated resistance regions, resistance islands, genomic islands, mobile genetic elements, and phylogenomic relationships within a global comparative framework. This study provides a comprehensive genomic characterization of two PDR ST308/O11 *P. aeruginosa* isolates recovered in Egypt and expands the currently available genomic data for understanding the resistance architecture, virulence-associated features, and genomic context of this globally disseminated high-risk lineage.

## Materials & methods

### Ethical statement

The study was approved by the Institutional Review Board (IRB) of the Military Medical Academy, Health and Preventive Medicine Institute, Ministry of Defense, Egypt (Approval No. 17-2025). All procedures involving human-derived samples were conducted in accordance with the ethical standards of the institutional research committee and the Declaration of Helsinki. Written informed consent was obtained from all participants prior to sample collection.

### Study design, setting, and population

This targeted genomic characterization study was conducted on two pandrug-resistant (PDR) *P. aeruginosa* isolates selected from a larger cohort of ventilator-associated pneumonia (VAP) patients following phenotypic screening and antimicrobial susceptibility testing.

The two strains were recovered from VAP patients hospitalized to the ICU at Al-Azhar University Hospital and Ain Shams University Hospital between November 2024 and March 2025. Both individuals displayed clinical signs of pneumonia during ICU admission and subsequently died. Demographic and clinical data of patients, encompassing age, sex, primary diagnosis, comorbidities, antibiotic administration, ICU duration, and survival outcomes, were extracted from clinical records in hospital documentation. The comprehensive workflow of this study is summarized in (Supplementary Figure S1).

### Study screening and isolate selection

A total of 100 clinical samples were collected from ventilator-associated pneumonia (VAP) patients admitted to the ICU during the study period. All samples were subjected to culture, bacterial identification, and antimicrobial susceptibility testing using disk diffusion and the VITEK® 2 compact system.

Among the collected isolates, Pseudomonas aeruginosa strains exhibiting the highest levels of antimicrobial resistance were identified based on their resistance profiles across multiple antimicrobial classes and VITEK-derived minimum inhibitory concentrations (MICs).

Two isolates demonstrating the most extensive resistance profiles, in addition to their association with fatal clinical outcomes, were selected for whole-genome sequencing and detailed genomic analysis.

### Isolation and identification of the organism

Endotracheal aspirate (ETA) specimens were cultured on blood agar, MacConkey agar, and nutrient agar plates (Oxoid, UK), and incubated aerobically at 35 ± 2 °C for 24 h.

Preliminary identification of bacterial isolates was performed using standard microbiological methods, including assessment of colony morphology, Gram staining, and a panel of biochemical tests, such as triple sugar iron (TSI) agar reactions, citrate utilization, urease production, motility testing, indole production, ornithine decarboxylation, and oxidase activity [13].

Species-level identification was subsequently confirmed using the automated VITEK^®^ 2 compact system (bioMérieux, France).

### Antimicrobial susceptibility testing

Antimicrobial susceptibility testing was conducted using the disk diffusion method, the VITEK^®^ 2 compact system, and the broth microdilution (BMD) method (for colistin susceptibility), in accordance with Clinical and Laboratory Standards Institute (CLSI) guidelines [14].

### Disk diffusion method (Kirby–Bauer)

The Kirby–Bauer disk diffusion method was performed on Mueller–Hinton agar (MHA) plates (Oxoid, UK). A bacterial suspension adjusted to the 0.5 McFarland standard was inoculated onto the agar surface, followed by the application of antibiotic disks. Plates were incubated aerobically at 37 °C for 18 h, and inhibition zones were interpreted according to CLSI criteria.

### VITEK^®^ 2 compact system

The automated VITEK^®^ 2 compact system (bioMérieux, France) was used to determine minimum inhibitory concentrations (MICs) for the tested antibiotics. MIC values were interpreted according to CLSI breakpoint standards.

### Broth microdilution method for colistin susceptibility

Colistin susceptibility was determined using the broth microdilution method in accordance with CLSI guidelines. Two-fold serial dilutions of colistin (colistimethate sodium; Merck Sharp & Dohme, UK) were prepared in cation-adjusted Mueller–Hinton broth, with final concentrations ranging from 128 µg/mL to 0.03 µg/mL.

The antimicrobial panel included antibiotics commonly used for the treatment of *Pseudomonas aeruginosa* infections, representing major antimicrobial classes such as β-lactams (including penicillins, cephalosporins, carbapenems, and monobactams), aminoglycosides, fluoroquinolones, and polymyxins. The tested antibiotics covered the clinically relevant categories recommended for *P. aeruginosa* susceptibility assessment.

The isolates exhibited resistance to all tested antimicrobial agents within these categories, which is consistent with a pandrug-resistant (PDR) phenotype based on the tested panel.

### DNA extraction and whole-genome sequencing

Genomic DNA was extracted from pure cultures of *P. aeruginosa* grown on tryptic soy agar (TSA) plates at 37 °C for 18–24 h. Several well-isolated colonies were suspended in phosphate-buffered saline (PBS, pH 7.4) and centrifuged at 10,000 × g for 5 min to obtain a bacterial pellet.

DNA extraction was performed using the QIAamp DNA Mini Kit (Qiagen, Hilden, Germany) according to the manufacturer’s instructions for Gram-negative bacteria. Briefly, bacterial pellets were subjected to enzymatic and chemical lysis, followed by DNA adsorption onto silica-based spin columns. After sequential washing steps to remove contaminants and residual proteins, genomic DNA was eluted in 50 µL of nuclease-free water and stored at − 20 °C until further analysis.

DNA concentration was quantified using the Qubit™ dsDNA High Sensitivity (HS) Assay Kit (Thermo Fisher Scientific, USA), while DNA purity was assessed using a NanoDrop 2000 spectrophotometer (Thermo Fisher Scientific, USA). DNA integrity was further evaluated by agarose gel electrophoresis prior to library preparation.

Library preparation was performed using the Oxford Nanopore Technologies Native Barcoding Kit 24 (SQK-NBD114.24) following the manufacturer’s protocol. Approximately 400 ng of high-quality genomic DNA was used as input for end-repair, dA-tailing, native barcode ligation, and library construction [10].

Sequencing was carried out on a PromethION 2 Solo platform (Oxford Nanopore Technologies, UK) using R10.4.1 flow cell chemistry. Basecalling was performed using Guppy v6.5.7 in high-accuracy mode, generating datasets with mean read quality scores exceeding Q20 for both isolates.

High-quality Nanopore reads were assembled de novo using Flye v2.9.5 and subsequently polished using Medaka v1.11.3 to improve consensus sequence accuracy. Assembly quality was assessed using QUAST v5.2.0 and BUSCO v5.6.1 prior to downstream genomic analyses.

The two clinical isolates were deposited in the NCBI Sequence Read Archive (SRA) under accession numbers SRR36023598 (BioSample SAMN53246146; Cairo2024_01) and SRR36023597 (BioSample SAMN53246147; Cairo2025_02).

### Multilocus sequence typing (MLST)

In silico multilocus sequence typing (MLST) was performed using the established *Pseudomonas aeruginosa* MLST scheme based on the allelic profiles of seven housekeeping genes (acsA, aroE, guaA, mutL, nuoD, ppsA, and trpE) [15]. Sequence types were assigned using publicly available MLST databases and bioinformatic tools. MLST was used to provide clonal lineage assignment and to support comparative genomic analyses.

### Average nucleotide identity (ANI) analysis

Average nucleotide identity (ANI) analysis was performed to assess whole-genome similarity between the study isolates and selected *Pseudomonas aeruginosa* reference genomes. Pairwise ANI values were calculated using FastANI v1.34, and reference genomes were ranked according to their mean ANI values relative to the study isolates. The resulting ANI matrix was used to evaluate genomic relatedness and species-level similarity among the analyzed genomes. Comparative visualization of ANI values was performed using the ggplot2 package in R [16]. Because ANI reflects overall genome-wide nucleotide similarity, it was used as a complementary measure to the higher-resolution core-genome SNP phylogenetic analysis.

### Mobile genetic elements (MGEs) analysis

The identification of mobile genetic elements (MGEs) was performed using the Center for Genomic Epidemiology (CGE) platform. MobileElementFinder v1.0.3 was used to detect insertion sequences, transposons, and other MGEs within the assembled genomes, based on curated databases and sequence similarity thresholds [17].

Detected MGEs were further analyzed in relation to antimicrobial resistance (AMR) genes to explore potential genetic associations and mobility. Co-localization analysis was performed to assess the spatial proximity of AMR genes and MGEs across the genome. Particular attention was given to the genomic context of key resistance determinants, including blaNDM-1, to evaluate their association with nearby mobile elements.

Regions exhibiting a high density of AMR genes and MGEs were considered putative AMR hotspots and were further examined for potential clustering patterns.

It is important to note that the identification of MGEs is dependent on database coverage and detection thresholds; therefore, the reported number of elements may represent a conservative estimate rather than a complete representation of all mobile elements present in the genomes.

### Core-genome SNP phylogenetic analysis

Comparative phylogenomic analysis was performed using the two study isolates together with 50 publicly available *Pseudomonas aeruginosa* reference genomes selected based on Mash distance [18]. Genome annotation was performed using Prokka v1.14.6 [19], followed by pangenome reconstruction and core-gene alignment generation using Panaroo v1.5.2 with the MAFFT aligner v7.490 [20].

Variable nucleotide positions were extracted from the core-gene alignment using snp-sites v2.5.1. The resulting SNP alignment was subsequently used for phylogenetic reconstruction.

Maximum-likelihood phylogenetic reconstruction was performed using IQ-TREE v3.1.1 under the GTR + ASC substitution model. Branch support was assessed using 2,000 ultrafast bootstrap replicates and 1,000 SH-aLRT replicates. The resulting phylogeny was midpoint-rerooted for visualization purposes [21].

Pairwise core-genome SNP distances were calculated to quantify genomic relatedness among isolates. The phylogenetic analysis was intended to assess comparative genomic relationships and should not be interpreted as evidence of direct epidemiological transmission.

### Detection of virulence genes

Virulence-associated genes were identified from the assembled genomes using ABRicate v1.0.1 within the Galaxy platform [22]. Genome assemblies were screened against the Virulence Factors Database (VFDB) to detect known virulence determinants [23].

Default alignment parameters were applied, and detected genes were filtered using a minimum nucleotide identity threshold of 90% and a minimum coverage threshold of 80% to ensure high-confidence predictions. Identified virulence-associated genes were subsequently categorized according to their functional roles, including secretion systems, iron acquisition, adhesion, and biofilm formation.

Because this analysis was based solely on genomic sequence data, the detected virulence-associated genes represent predicted virulence potential rather than confirmed phenotypic expression. Therefore, the presence of these genes should not be interpreted as direct evidence of virulence activity or clinical impact.

### Comparative genomic visualization and data representation

Linear genome maps were generated using Proksee to visualize the genomic context and spatial distribution of resistance determinants and mobile genetic elements across the assemblies. Radar plots were constructed to summarize virulence-associated gene categories identified in the two study isolates. Additional graphical representations were generated using the ggplot2 v3.5.1 package in R [24].

Circular genome maps were generated using Proksee to provide an overview of genome organization, genome annotation features, antimicrobial resistance genes, virulence-associated genes, and GC-content characteristics of the assembled genomes [25].

Comparative genomic visualization was performed to summarize genome structure and to illustrate the distribution of antimicrobial resistance (AMR) genes, virulence-associated genes, and mobile genetic elements (MGEs) across the studied *P. aeruginosa* isolates and selected reference genomes.

Comparative pan-genome visualization was generated from the Panaroo output to illustrate the distribution of core, soft-core, shell, and cloud gene clusters across the analyzed genomes.

These visualization approaches were used to support comparative interpretation of genomic features identified through bioinformatic analyses and do not constitute functional validation of the detected genetic determinants.

### Chromosomal resistance mutation analysis

Chromosomal mutations associated with antimicrobial resistance were investigated using AMRFinderPlus v3.12.8 and comparative analysis against the *Pseudomonas aeruginosa* PAO1 reference genome. Particular attention was given to genes commonly associated with resistance phenotypes, including *gyrA*, *gyrB*, *parC*, *parE*, *oprD*, *mexR*, *nalC*, *nalD*, *mexZ*, *ampD*, *ampR*, *pmrA*, *pmrB*, *phoP*, and *phoQ*. Identified variants were evaluated against curated antimicrobial resistance databases and published literature to distinguish established resistance-associated mutations from variants of uncertain significance [21].

### Integron and resistance island analysis

Class 1 integrons were identified using IntegronFinder v2.0 by detecting integron integrases (intI), attC recombination sites, and gene cassette arrays. Integrase sequences were subsequently confirmed as IntI1 by BLASTP and BLASTN searches against curated class 1 integron integrase reference sequences. Promoter regions upstream of the integrase genes were manually examined, and Pc promoter variants were assigned according to the conserved − 35 and − 10 hexamer motifs described by Jové et al. Complete class 1 integrons and In0 structures were classified based on the presence or absence of attC recombination sites and gene cassette arrays. Resistance islands were further characterized by integrating genomic island predictions, resistance gene localization, and the genomic context of clinically important antimicrobial resistance determinants, including blaNDM-1, together with associated mobile genetic elements and neighboring resistance genes [26].

### Plasmid detection and chromosomal localization analysis

Plasmid replicons were identified using PlasmidFinder v2.1.6 with the default parameters and a minimum nucleotide identity threshold of 95%. To determine the genomic origin of assembled contigs, MOB-suite v3.1.9 (MOB-recon) was used to classify contigs as chromosome- or plasmid-derived based on mobility-associated features and plasmid reference databases. The chromosomal localization of clinically relevant antimicrobial resistance determinants, including blaNDM-1, class 1 integrons, and major resistance islands, was subsequently evaluated by integrating MOB-recon contig classifications with genome annotation and resistance island analyses [27].

### Genomic island analysis

Putative genomic islands were identified using sequence composition-based and mobility-associated features, including deviations in GC content, mobility-associated genes, and genomic signatures indicative of horizontal gene transfer. Predicted genomic islands were subsequently examined for the presence of antimicrobial resistance genes, virulence-associated determinants, and mobile genetic elements. Islands containing clinically relevant resistance determinants or virulence factors were classified according to their predominant genetic content and biological significance [28].

## Results

Two PDR *P. aeruginosa* strains were obtained from ETA of two patients diagnosed with VAP who later died in the ICU at Al-Azhar University Hospital and Ain Shams University Hospital. The clinical and demographic data of the two patients are listed in (Table 1).

**Table 1:** Clinical characteristics of patients from whom *P. aeruginosa* isolates were obtained

Clinical variable	Cairo2024_01 (SRR36023598)	Cairo2025_02 (SRR36023597)
Sex	Female	Female
Age (years)	58	59
Primary diagnosis	Pneumonia with acute respiratory failure	Pneumonia with respiratory distress
Comorbidities	DM, HTN, IHD, hypothyroidism, ESRD	DM, HTN, IHD, AF, stroke
Date of ICU admission	16 April 2025	21 June 2025
Date of VAP diagnosis	20 April 2025	26 June 2025
Date of ETA sample collection	20 April 2025	26 June 2025
ICU day at isolate recovery	ICU day 4	ICU day 5
Length of ICU stay (days)	3	10
Antimicrobial therapy before isolation	Meropenem, Levofloxacin	Meropenem, Teicoplanin
Antimicrobial therapy after susceptibility results	Patient died before susceptibility results became available	Colistin, Meropenem
Clinical evidence supporting VAP	Clinical diagnosis of VAP with positive endotracheal aspirate (ETA) culture	Clinical diagnosis of VAP with positive endotracheal aspirate (ETA) culture
Outcome	Died	Died

*DM* Diabetes mellitus, *HTN* Hypertension, *IHD* Ischemic heart disease, *ESRD* End-stage renal disease, *AF* Atrial fibrillation

The two *P. aeruginosa* isolates exhibited resistance to all antimicrobial agents tested using disk diffusion, the VITEK 2 compact system, and broth microdilution for colistin. The tested antimicrobial panel included representatives from major antibiotic classes, including β-lactams (piperacillin, piperacillin/tazobactam, ticarcillin, ticarcillin/clavulanic acid, ceftazidime, cefepime, imipenem, meropenem, doripenem, and aztreonam), aminoglycosides (gentamicin, tobramycin, netilmicin, and amikacin), fluoroquinolones (ciprofloxacin, levofloxacin, norfloxacin, ofloxacin, and gatifloxacin), and polymyxins (colistin).

The observed resistance profile was consistent with pandrug resistance based on the antimicrobial agents tested in this study.

### Genome sequencing, assembly characteristics and mlst

Whole-genome sequencing generated high-quality draft assemblies for both *P. aeruginosa* isolates. Raw sequencing datasets were deposited in the NCBI Sequence Read Archive (SRA) under accession numbers SRR36023598 (BioSample SAMN53246146; Cairo2024_01) and SRR36023597 (BioSample SAMN53246147; Cairo2025_02).

The assembly of Cairo2024_01 consisted of two contigs with a total genome size of 6,764,468 bp and a GC content of 66.15%, whereas Cairo2025_02 was assembled into four contigs with a total genome size of 6,995,336 bp and a GC content of 65.96%. Both assemblies exhibited high completeness, with BUSCO scores of 99.5%, indicating near-complete genome recovery suitable for downstream comparative genomic analyses. Sequencing statistics, assembly metrics, and annotation characteristics are summarized in (Table 2).

**Table 2:** Genome characteristics of Cairo2024_01 and Cairo2025_02 strains

Metric	Cairo2024_01 (SRR36023598)	Cairo2025_02 (SRR36023597)
Mean read quality (Q score)	20.11	20.16
Total reads	288,121	283,556
Total bases	1.61 Gb	1.41 Gb
Read N50 (bp)	14,923	10,780
Genome size (bp)	6,764,468	6,995,336
Number of contigs	2	4
Contig N50 (bp)	6,764,468	6,995,336
GC content (%)	66.15	65.96
BUSCO completeness (%)	99.5	99.5
Protein-coding sequences (CDSs)	6,370	6,659
tRNA genes	66	69
rRNA genes	12	12

Genome annotation identified 6,370 protein-coding sequences (CDSs), 66 tRNA genes, and 12 rRNA genes in Cairo2024_01, while Cairo2025_02 contained 6,659 CDSs, 69 tRNA genes, and 12 rRNA genes. Despite minor differences in genome size and accessory gene content, both genomes exhibited highly similar structural organization and functional annotation profiles (Supplementary Figure S2A–B). consistent with their close evolutionary relationship.

In silico multilocus sequence typing (MLST) assigned both isolates to sequence type ST308. This lineage is recognized as a globally disseminated high-risk clone frequently associated with healthcare-associated infections, multidrug resistance, and international transmission events. The shared ST308 designation, together with the overall genomic similarity observed between the two isolates, suggested a highly conserved clonal background and provided the framework for subsequent comparative genomic analyses.

### Antimicrobial resistance genes (AMR)

Among the 100 clinical *Pseudomonas aeruginosa* isolates screened in this study, antimicrobial susceptibility testing revealed a high prevalence of multidrug resistance (Supplementary Table S1). The two isolates selected for whole-genome sequencing represented the most extensively resistant isolates identified in the cohort and were therefore subjected to detailed comparative genomic analysis.

Comparative resistome analysis using CARD-RGI revealed highly similar antimicrobial resistance gene profiles in both *Pseudomonas aeruginosa* isolates (Cairo2024_01 and Cairo2025_02). Resistance determinants spanning multiple antimicrobial classes were identified, indicating a broadly conserved resistome architecture between the two genomes. The overall resistome composition and CARD-RGI classification profiles are shown in (Supplementary Figure S3A–B).

Resistance to β-lactam antibiotics was associated with the presence of *blaNDM-1*, *blaOXA-10*, *blaOXA-488*, and the chromosomally encoded cephalosporinase *PDC-19*. Multiple efflux-associated systems, including MexAB-OprM, MexEF-OprN, MexCD-OprJ, and MexXY, were also detected in both isolates.

Additional resistance determinants were identified for fluoroquinolones (*qnrVC1*), aminoglycosides (*APH(3′)-IIb*, *APH(6)-Id*, *APH(3′′)-Ib*, and *rmtF*), phenicols (*floR* and *catB7*), sulfonamides (*sul1* and *sul2*), macrolide/streptogramin antibiotics (*msrE*), trimethoprim (*dfrB5*), and fosfomycin (*FosA*) (Tables 3 and 4).

**Table 3:** Major antimicrobial resistance genes identified in both *Pseudomonas aeruginosa* isolates (Cairo2024_01 and Cairo2025_02)

Strain	β-lactam resistance genes	Aminoglycoside resistance genes	Amphenicol resistance genes	Macrolide/Streptogramin resistance genes	Quinolone resistance genes	Folate pathway antagonist resistance genes	Aminocyclitol resistance genes
Cairo2024_01 & Cairo2025_02	*blaPAO*, *blaNDM-1*, *blaOXA-488*, *blaOXA-10*, *PDC-19*	*aph(3’)-IIb*, *APH(3’’)-Ib*, *rmtF*, *aph(6)-Id*, *aac(3)-Id*	*floR*, *catB7*	*msr(E)*	*qnrVC1*	*sul1*, *sul2*, *dfrB5*	*aadA10*, *FosA*

**Table 4:** High-confidence antimicrobial resistance determinants identified in both *Pseudomonas aeruginosa* isolates (Cairo2024_01 and Cairo2025_02) based on CARD-RGI analysis

RGI Criteria	Gene (ARO term)	Drug class affected	Resistance mechanism
Perfect	*blaNDM-1*	Carbapenems, β-lactams	Antibiotic inactivation
Perfect	*blaOXA-10*	Cephalosporins, penicillins	Antibiotic inactivation
Perfect	*blaOXA-488*	Penicillins	Antibiotic inactivation
Perfect	*PDC-19*	Cephalosporins, carbapenems	Antibiotic inactivation
Perfect	*qnrVC1*	Fluoroquinolones	Antibiotic target protection
Perfect	*sul1*	Sulfonamides	Antibiotic target replacement
Perfect	*sul2*	Sulfonamides	Antibiotic target replacement
Perfect	*rmtF*	Aminoglycosides	Antibiotic target modification
Perfect	*APH(3’)-IIb*	Aminoglycosides	Antibiotic inactivation
Perfect	*APH(6)-Id*	Aminoglycosides	Antibiotic inactivation
Perfect	*APH(3’’)-Ib*	Aminoglycosides	Antibiotic inactivation
Perfect	*msrE*	Macrolides, streptogramins	Antibiotic target protection
Perfect	*catB7*	Phenicols	Antibiotic inactivation
Perfect	*floR*	Phenicols	Antibiotic efflux
Perfect	*FosA*	Phosphonic acid (fosfomycin)	Antibiotic inactivation

*RGI* Resistance Gene Identifier, *ARO* Antibiotic Resistance Ontology

perfect and strict (i.e.; with high confidence and probability)

*Only minimal variation was observed between the two isolates with respect to resistance gene content and resistance mechanism distribution. No substantial differences were detected in the major antimicrobial classes affected or the overall resistome composition, supporting the presence of a highly conserved resistance gene repertoire in both ST308 isolates.

### Chromosomal resistance mutations

Analysis of chromosomal resistance-associated loci identified multiple high-confidence mutations in both *P. aeruginosa* isolates (Table 5). The mutation profiles were identical in Cairo2024_01 and Cairo2025_02.

**Table 5:** High-confidence chromosomal resistance-associated mutations identified in the two *Pseudomonas aeruginosa* ST308 isolates

Gene	Amino acid change	Resistance association	phenotype
*gyrA*	T83I	QRDR mutation	Fluoroquinolone resistance
*parC*	S87L	QRDR mutation	Fluoroquinolone resistance
*oprD*	Q402HfsTer99	Porin disruption	Carbapenem resistance
*nalC*	G71E	Efflux regulation	Multidrug resistance
*nalC*	S209R	Efflux regulation	Multidrug resistance
*nalD*	S82AfsTer18	Efflux regulation	Multidrug resistance
*mexR*	V126E	Putative efflux derepression	Multidrug resistance
*pmrB*	V15I	PmrAB pathway alteration	Reduced colistin susceptibility

Mutations were identified in both Cairo2024_01 and Cairo2025_02. Resistance associations were assigned based on published evidence and curated antimicrobial resistance databases

*QRDR* Quinolone resistance-determining region

Both isolates carried the quinolone resistance-determining region (QRDR) mutations *gyrA* T83I and *parC* S87L. A truncating mutation in the outer membrane porin *oprD* (Q402HfsTer99) was also detected in both genomes.

Additional mutations were identified in regulators of the MexAB-OprM efflux system, including *nalC* (G71E and S209R), *nalD* (S82AfsTer18), and *mexR* (V126E). Furthermore, both isolates carried the *pmrB* substitution V15I.

Several additional variants were identified in resistance-associated loci, including *parE*, *ampR*, *mexZ*, and *oprD*, and were classified as variants of uncertain significance (Supplementary Table S4).

A complete summary of high-confidence resistance-associated mutations is provided in (Table 5).

### Integron-associated resistance modules

Analysis of integron-associated regions identified two complete class 1 integrons and one In0 structure in each *Pseudomonas aeruginosa* isolate, whereas no class 2 or class 3 integrons were detected. The integron integrase (intI) genes identified by IntegronFinder were confirmed as class 1 integron integrases (IntI1) by BLASTP and BLASTN analyses against curated reference sequences. The first complete integron contained the *aac(6’)-II–dfrB5–aac(3)-Id* cassette array associated with three attC recombination sites, whereas the second complete integron carried *qnrVC1–aadA11* with two attC sites. The In0 structure contained an IntI1 integrase but lacked attC sites and gene cassette arrays. Promoter analysis identified a PcH2 promoter in the first complete integron and PcW promoters in the second complete integron and the In0 structure. A summary of the structural characteristics of the identified class 1 integrons is presented in (Table 6). Several clinically relevant resistance genes, including *blaOXA-10*,* rmtF*,* blaPAC-1*,* qacEΔ1*, and *sul1*, were associated with these integron regions, whereas *blaNDM-1*,* msrE*,* floR*, and *sul2* were located outside the identified integron structures. The overall integron architecture was highly conserved between Cairo2024_01 and Cairo2025_02. 

**Table 6:** Detailed characterization of class 1 integrons identified in the two Egyptian ST308 isolates

Feature	Integron 1	Integron 2	In0 structure
Structure type	Complete class 1 integron	Complete class 1 integron	In0
Integrase	IntI1	IntI1	IntI1
Integrase confirmation	BLASTP/BLASTN (99.7–100% identity; 100% coverage)	BLASTP/BLASTN (100% identity; 100% coverage)	BLASTP/BLASTN (100% identity; 100% coverage)
Pc promoter	PcH2	PcW	PcW
Promoter classification	Jové et al. (2010) − 35/−10 hexamers	Jové et al. (2010) − 35/−10 hexamers	Jové et al. (2010) − 35/−10 hexamers
P2 promoter	Not detected	Not detected	Not detected
attC sites	3	2	None
Gene cassette organization	aac(6′)-II → dfrB5 → aac(3)-Id	qnrVC1 → aadA11	No cassette array (In0)
Distribution	Present in both isolates	Present in both isolates	Present in both isolates

### Resistance islands and genomic islands

Genomic island analysis identified multiple regions associated with antimicrobial resistance and virulence in both *Pseudomonas aeruginosa* isolates. The overall genomic island architecture was highly conserved between Cairo2024_01 and Cairo2025_02 and included resistance islands, resistance–virulence islands, virulence-associated islands, and additional genomic islands (Table 7).

**Table 7:** Major genomic islands identified in the Egyptian ST308 isolates

Island ID	Island type	Key genetic content
GI_14	Resistance island	*sul2*, *floR*, Tn5393, *aph(3’’)-Ib*
GI_26	Resistance island	*qnrVC1*, *aadA11*, *aac(6’)-II*, *dfrB5*, *aac(3)-Id*
GI_27	Resistance island	*sul1*, *rmtF*, *blaOXA-10*, *blaPAC-1*, *qacEΔ1*
GI_32	Resistance–virulence island	*exoU*-containing genomic region
GI_42	Resistance island	*blaNDM-1*, *ble*, *msrE*, ISVsa3

Only the major resistance- and virulence-associated genomic islands are shown. Complete genomic island annotations, coordinates, and classifications are provided in (Supplementary Table S5)

Several major resistance-associated genomic islands were identified. A genomic island located at approximately 1.89–1.93 Mb (GI_14) contained *sul2*, *floR*, *aph(3’’)-Ib*, and the transposon Tn5393. A second resistance-associated island GI_26 harbored a complete class 1 integron containing the *qnrVC1–aadA11* cassette array together with additional aminoglycoside and trimethoprim resistance determinants. Another multidrug resistance island GI_27 contained an In0-associated class 1 integron module carrying *sul1*,* rmtF*,* blaOXA-10*,* blaPAC-1*, and *qacEΔ1*.

A distinct resistance island (GI_42) carried *blaNDM-1* together with *ble*, *msrE*, and the insertion sequence ISVsa3. This genomic region was separate from the integron-associated resistance modules identified elsewhere in the chromosome.

In addition to resistance-associated islands, the cytotoxic type III secretion system effector *exoU* was localized within a predicted genomic island (GI_32). The genomic organization of this region was conserved in both isolates.

Overall, Cairo2024_01 contained five resistance islands, nine resistance–virulence islands, twenty-three virulence-associated islands, and five additional genomic islands, whereas Cairo2025_02 contained six resistance islands, eight resistance–virulence islands, twenty virulence-associated islands, and six additional genomic islands.

A summary of the major genomic islands identified in both isolates is presented in (Table 7), while complete genomic island annotations and coordinates are provided in (Supplementary Table S5).

### Mobile genetic elements

Mobile genetic elements (MGEs) were identified through sequence comparison against a curated database comprising insertion sequences, transposons, and related mobile DNA elements. A limited number of MGEs were detected in both *P. aeruginosa* genomes, with most elements occurring at corresponding genomic locations, indicating a highly conserved mobilome architecture.

Several antimicrobial resistance determinants were found in association with mobile genetic elements. The *blaNDM-1* resistance island contained the IS91-family insertion sequence ISVsa3, whereas the *floR–sul2* resistance island was associated with the transposon Tn5393. In addition, the multidrug resistance region carrying *sul1*, *rmtF*, *blaOXA-10*, and *blaPAC-1* was located adjacent to the Tn3-family transposon TnAs1. The *qnrVC1* cassette array was associated with a nearby XerC recombinase.

No plasmid sequences were detected using the applied analytical approaches. Overall, both isolates exhibited highly similar MGE distributions and AMR–MGE association patterns, consistent with the extensive conservation observed across their resistance-associated genomic regions.

The genomic distribution of mobile genetic elements and their relationship to antimicrobial resistance determinants are illustrated in (Supplementary Figure S4A–B). Detailed MGE annotations are provided in (Supplementary Table S6).

### Plasmid detection and chromosomal localization

Plasmid analysis using PlasmidFinder v2.1.6 detected no plasmid replicons in either Cairo2024_01 or Cairo2025_02. Consistently, MOB-suite v3.1.9 (MOB-recon) classified all assembled contigs as chromosomal, with no plasmid-derived contigs identified. Furthermore, the *blaNDM-1*-containing resistance island, the two complete class 1 integrons, the In0 structure, and the major antimicrobial resistance islands were all localized to chromosomal regions. Although several mobility-associated genes were identified within chromosomal resistance-associated regions, no autonomous plasmids were detected. These findings indicate that the antimicrobial resistance determinants characterized in both isolates are chromosomally encoded and that resistance dissemination is primarily associated with chromosomal mobile genetic elements rather than plasmids.

### Core-genome SNP phylogenetic analysis

To investigate the evolutionary relationships of the Egyptian isolates within a global context, a comparative core-genome phylogenomic analysis was performed using Cairo2024_01, Cairo2025_02, and 50 publicly available *Pseudomonas aeruginosa* genomes selected based on Mash distance. All genomes ranked by core SNP distance to Cairo2024_01 and Cairo2025_02 are illustrated in (Supplementary table S3).

Pan-genome reconstruction identified 12,765 gene clusters, including 5,085 core genes shared among all analyzed genomes. Alignment of the core genome yielded 144,617 informative single nucleotide polymorphisms (SNPs), which were subsequently used for maximum-likelihood phylogenetic reconstruction (Supplementary Figure S5). 

Both Egyptian isolates clustered within the internationally distributed ST308 lineage and formed a well-supported monophyletic subclade. Pairwise comparison identified only 74 core-genome SNP differences between Cairo2024_01 and Cairo2025_02, indicating a high degree of genomic similarity. The closest phylogenetic neighbors of the Egyptian isolates were ST308 genomes originating from Argentina and Canada. Despite their close phylogenetic placement, comparative genomic analyses demonstrated differences in accessory resistance gene content, particularly with respect to carbapenemase-associated regions. Metadata for all genomes included in the phylogenomic analysis are illustrated in (Supplementary table S2)

Overall, the phylogenomic analysis demonstrated that the Egyptian isolates belong to a globally disseminated ST308 lineage while maintaining a highly conserved genomic background relative to other international representatives.

### Virulence-associated determinants

Virulence gene profiling revealed highly similar virulence-associated repertoires in Cairo2024_01 and Cairo2025_02. Identified genes were distributed across multiple functional categories associated with *Pseudomonas aeruginosa* pathogenicity, including iron acquisition, biofilm formation, adhesion, secretion systems, and phenazine biosynthesis.

Genes involved in pyoverdine-mediated iron acquisition (*pvdA* and *pvdS*), alginate biosynthesis and biofilm formation (*algD*, *algU*, *algR*, *mucA*, and *mucB*), and pili-associated functions (*pilB*, *pilY1*, and *pilR*) were detected in both isolates. In addition, both genomes carried phospholipase (*plcH*) and phenazine biosynthesis genes (*phzM*, *phzS*, and *phzH*).

Both isolates harbored components of the type III secretion system (T3SS), including *exoU* and *exoT*, as well as type VI secretion system (T6SS) genes (*vgrG1a*, *hsiJ1*, *dotU1*, and *icmF1*). No substantial differences in virulence gene content were observed between Cairo2024_01 and Cairo2025_02, consistent with their close phylogenetic relationship and overall genomic similarity.

A detailed summary of virulence-associated genes, including gene coverage, nucleotide identity, and predicted functions, is provided in (Supplementary Table S7).

### Comparative genomic characteristics of Cairo2024_01 and Cairo2025_02

Comparative analyses demonstrated a high degree of similarity between Cairo2024_01 and Cairo2025_02 with respect to genome architecture, antimicrobial resistance determinants, virulence-associated genes, chromosomal resistance mutations, genomic islands, and mobile genetic element profiles. Both isolates belonged to ST308 and exhibited pandrug-resistant phenotypes.

Pan-genome analysis revealed highly similar gene presence–absence patterns between the two isolates (Supplementary Figure S6), whereas comparative heatmap analysis demonstrated closely related distributions of antimicrobial resistance genes, virulence-associated determinants, and insertion sequence families relative to the international ST308 dataset (Supplementary Figure S7). These findings are consistent with the close genomic relationship observed in the core-genome SNP phylogeny.

### Average nucleotide identity (ANI) analysis

Average nucleotide identity (ANI) analysis demonstrated that both *Pseudomonas aeruginosa* isolates (Cairo2024_01 and Cairo2025_02) shared consistently high ANI values (> 99%) across all selected reference genomes, remaining above the accepted species delineation threshold of 95%.

The ANI profiles of the two isolates were highly similar across the ranked reference dataset, with only minor variation observed between them. The mean inter-isolate difference across all comparisons was 0.0712% ANI, indicating limited genome-wide divergence.

The highest ANI values were observed relative to ST308 reference genomes originating from Argentina and Canada, consistent with the phylogenetic placement of both Egyptian isolates within the ST308 lineage. High nucleotide similarity was also observed across multiple additional international ST308 genomes included in the comparative dataset.

Overall, ANI analysis confirmed the close genomic relatedness of Cairo2024_01 and Cairo2025_02 and supported their placement within a globally distributed ST308 lineage.

## Discussion

*Pseudomonas aeruginosa* remains one of the most clinically challenging pathogens associated with ventilator-associated pneumonia (VAP), particularly among critically ill patients receiving prolonged antimicrobial therapy and mechanical ventilation. The remarkable adaptive capacity of this organism, combined with its ability to accumulate resistance determinants and virulence-associated traits, has facilitated the emergence of globally disseminated high-risk clones that represent a major threat to healthcare systems worldwide [1–7]. In the present study, whole-genome sequencing and comparative genomic analyses revealed that two pandrug-resistant (PDR) *P. aeruginosa* isolates recovered from fatal VAP cases in Egypt belonged to the internationally distributed ST308/O11 lineage and possessed a complex genomic architecture characterized by resistance islands, integron-associated multidrug resistance modules, chromosomal resistance mutations, and virulence-associated determinants.

Comparative phylogenomic analysis demonstrated that both Egyptian isolates belonged to ST308 and formed a highly conserved sublineage separated by only 74 core-genome SNPs. ST308 has emerged as a globally distributed high-risk clone increasingly associated with healthcare-associated infections and extensive antimicrobial resistance [5, 7]. NDM-producing ST308 isolates reported from Singapore and Greece carried resistance island structures resembling those observed in the present study, supporting repeated acquisition of carbapenem-resistance modules within the ST308 lineage [29, 30]. Similarly, reports from multiple geographic regions demonstrate the global dissemination of ST308 despite substantial variation in accessory resistance content. Together, these observations indicate that ST308 possesses a conserved chromosomal framework capable of independently acquiring distinct resistance islands in different geographical settings.

The international dissemination of ST308 has been accompanied by substantial diversification of the accessory genome. Although the Egyptian isolates clustered most closely with ST308 genomes originating from Argentina and Canada, comparative genomic analyses revealed important differences in resistance gene content. Most notably, neither of these closely related genomes carried the *blaNDM-1* resistance island identified in the Egyptian isolates. This finding suggests that acquisition of the NDM-associated island may have occurred after divergence from their nearest currently available relatives and illustrates the substantial contribution of horizontal gene transfer to the evolution of ST308 populations [5, 7, 31]. Such observations highlight the dynamic nature of accessory genome evolution, whereby closely related strains may exhibit major differences in resistance potential despite sharing highly conserved chromosomal backgrounds.

The extensive antimicrobial resistance phenotype observed in the present study appears to result from the cumulative action of multiple complementary resistance mechanisms rather than dependence on a single determinant. Both genomes carried the metallo-β-lactamase gene *blaNDM-1* together with *blaOXA-10*, *blaOXA-488*, and the chromosomal cephalosporinase *PDC-19*. However, acquired resistance genes alone cannot fully explain the extensive resistance phenotype observed in these isolates.

The identification of the well-characterized quinolone resistance-determining region (QRDR) mutations *gyrA* T83I and *parC* S87L provides a strong genetic basis for fluoroquinolone resistance [26], whereas the truncating *oprD* mutation is consistent with reduced carbapenem permeability. In addition, mutations affecting *nalC*, *nalD*, and *mexR* suggest potential dysregulation of the MexAB-OprM efflux system, while the *pmrB* V15I substitution may contribute to reduced polymyxin susceptibility. Collectively, these findings indicate that resistance in the Egyptian isolates is supported by a multilayered genomic architecture involving acquired resistance genes, target-site alterations, porin disruption, and putative efflux-associated mechanisms.

The coexistence of the QRDR mutations *gyrA* T83I and *parC* S87L in both Egyptian ST308 isolates is consistent with a mutation combination that has previously been associated not only with high-level fluoroquinolone resistance but also with enhanced bacterial fitness and the successful dissemination of multidrug-resistant lineages. Interestingly, neither isolate harbored a mutation affecting the *gyrA* D87 residue, which has been associated with a fitness cost and reduced virulence in certain multidrug-resistant *P. aeruginosa* lineages. The absence of *gyrA* D87 substitutions in the present isolates may therefore have contributed to the maintenance of a highly successful genomic background while preserving the multidrug-resistant phenotype. However, the effects of the identified QRDR mutations on bacterial fitness, virulence, and transmissibility were not experimentally evaluated in the present study and therefore cannot be inferred from genomic data alone [32–34].

An important finding of this study was the identification of multiple integron-associated multidrug resistance modules. Integrons are recognized as major drivers of antimicrobial resistance evolution because they facilitate the capture, accumulation, and expression of diverse resistance gene cassettes. The first integron-associated cassette array contained *aac(6′)-II*, *dfrB5*, *aac(3)-Id*, *qnrVC1*, and *aadA11*, whereas a second resistance module contained *blaOXA-10*, *rmtF*, *blaPAC-1*, *qacEΔ1*, and *sul1*. Together, these structures provide resistance determinants targeting aminoglycosides, fluoroquinolones, sulfonamides, trimethoprim, disinfectants, and β-lactam antibiotics. The coexistence of multiple resistance cassettes within conserved integron-associated regions emphasizes the importance of integrons as reservoirs for multidrug resistance and highlights their contribution to the successful adaptation of hospital-associated *P. aeruginosa* clones [28].

The genomic context of *blaNDM-1* provides additional insight into the evolutionary history of these isolates. Unlike several other resistance determinants that were embedded within integron-associated regions, *blaNDM-1* was located within a distinct resistance island containing *ble*, *msrE*, and the IS91-family insertion sequence ISVsa3. The organization of the *blaNDM-1–ble–msrE* region observed in the Egyptian isolates resembles resistance structures previously reported in internationally disseminated NDM-producing *P. aeruginosa* high-risk clones [29, 30], further supporting the role of mobile genetic elements in the global spread of carbapenem resistance. The physical separation of the *blaNDM-1* island from the major integron-associated resistance modules suggests that multiple independent horizontal acquisition events may have contributed to the assembly of the present resistance architecture. Such stepwise accumulation of mobile resistance elements likely represents an important evolutionary mechanism underlying the emergence of PDR phenotypes.

An additional important finding was the absence of detectable autonomous plasmids despite the extensive resistome observed in both isolates. PlasmidFinder and MOB-suite analyses failed to identify plasmid replicons or plasmid-derived contigs, indicating that the major antimicrobial resistance determinants, including *blaNDM-1* and the identified class 1 integrons, were chromosomally localized. This chromosomal organization suggests that the multidrug-resistant phenotype is primarily maintained through chromosomal mobile genetic elements, including resistance islands and integron-associated regions, rather than plasmid-mediated carriage. Similar chromosomal integration of clinically important resistance determinants has been reported in high-risk *P. aeruginosa* lineages and may contribute to the stable maintenance of antimicrobial resistance under sustained selective pressure. However, the potential impact of this genomic organization on bacterial fitness or transmissibility was not experimentally evaluated in the present study [27].

Genomic island analysis further demonstrated that resistance determinants were concentrated within several discrete chromosomal regions rather than being randomly distributed throughout the genome. Multiple resistance-associated islands carrying *blaNDM-1*, *qnrVC1*, *sul1*, *rmtF*, *floR*, and additional resistance determinants were identified in both isolates. The clustering of resistance genes within genomic islands has been reported in numerous high-risk *P. aeruginosa* lineages and is considered an efficient mechanism for maintaining functionally linked resistance determinants under selective antimicrobial pressure [35]. The high conservation of these genomic islands between Cairo2024_01 and Cairo2025_02 further supports the close evolutionary relationship of the two isolates.

Beyond antimicrobial resistance, both genomes carried a highly conserved repertoire of virulence-associated genes involved in iron acquisition, biofilm formation, secretion systems, and host interaction. Of particular interest was the detection of the type III secretion system effector *exoU*. Previous investigations have associated *exoU*-positive strains with increased cytotoxicity, enhanced tissue damage, severe disease manifestations, and poor clinical outcomes. Although genomic data alone cannot establish virulence expression, the coexistence of *exoU* with extensive antimicrobial resistance is clinically significant because it represents the convergence of virulence and resistance traits within the same lineage. The localization of *exoU* within a predicted genomic island further supports the role of horizontal gene transfer in shaping the virulence potential of these isolates.

An additional noteworthy feature of the Egyptian isolates is their assignment to the O11 serogroup. O11 is among the most frequently reported serogroups associated with epidemic and high-risk *P. aeruginosa* lineages and has been linked to successful hospital adaptation, persistence, and dissemination. The identification of an O11-ST308 background in the present study therefore places the Egyptian isolates within a genomic framework commonly associated with successful healthcare-adapted clones. The coexistence of the O11 serogroup, extensive antimicrobial resistance, and the cytotoxic effector *exoU* may further contribute to the epidemiological success and persistence of these isolates within hospital settings [31].

The combined presence of chromosomal resistance mutations, integron-associated multidrug resistance modules, resistance islands carrying *blaNDM-1*, and virulence-associated determinants highlights the adaptive capacity of the ST308 lineage. Rather than relying on a single resistance mechanism, these isolates possess multiple overlapping genomic strategies that collectively enhance survival under antimicrobial pressure while maintaining substantial pathogenic potential. Such genomic configurations are characteristic of successful high-risk clones capable of persistence, dissemination, and long-term establishment within healthcare environments.

Importantly, the present genomic investigation was embedded within a larger cohort comprising 100 VAP-associated clinical isolates collected from multiple healthcare institutions. The two sequenced isolates represented the most extensively resistant isolates identified during cohort-level screening and were associated with fatal clinical outcomes. Consequently, although whole-genome sequencing was performed on only two isolates, the findings should be interpreted within the broader context of antimicrobial resistance surveillance among VAP-associated *P. aeruginosa* circulating in Egyptian healthcare settings.

Several limitations should be acknowledged. Only two isolates were subjected to whole-genome sequencing, and therefore the findings may not fully represent the diversity of *P. aeruginosa* circulating in Egyptian healthcare settings. In addition, whole-genome sequencing was performed using Oxford Nanopore long-read technology without complementary Illumina short-read sequencing. Although recent Nanopore chemistry and high-accuracy basecalling substantially improve consensus accuracy, residual sequencing errors, particularly small insertions/deletions and single-nucleotide variants, may influence fine-scale variant detection. To minimize this effect, genome assemblies were generated using Flye, polished with Medaka, and evaluated using QUAST and BUSCO, providing high-quality genome assemblies suitable for comparative genomic analyses. Nevertheless, future studies incorporating hybrid long- and short-read sequencing could further improve the accuracy of SNP-level analyses and small variant detection. Furthermore, functional validation of resistance mechanisms, virulence factor expression, and horizontal transfer potential was beyond the scope of this study. Future investigations incorporating larger genomic datasets, complete genome assemblies, transcriptomic analyses, and phenotypic validation experiments will be valuable for further understanding the emergence and dissemination of ST308 lineages in Egypt and neighboring regions.

Overall, this study indicates that fatal VAP-associated ST308/O11 *P. aeruginosa* isolates identified through cohort-level screening of 100 clinical VAP-associated isolates possess a highly conserved genomic backbone enriched with integron-associated multidrug resistance modules, chromosomal resistance mutations, resistance islands carrying *blaNDM-1*, and virulence-associated determinants including *exoU*. Comparative analyses further indicate that the Egyptian isolates are embedded within a globally disseminated ST308 lineage while harboring accessory resistance elements absent from their closest international relatives. The convergence of these features highlights the continuing evolution of high-risk *P. aeruginosa* clones and underscores the importance of genomic surveillance for monitoring the emergence and spread of clinically significant lineages.

## Conclusion

This study provides a comprehensive genomic characterization of two highly resistant *Pseudomonas aeruginosa* isolates associated with fatal ventilator-associated pneumonia (VAP) in Egypt. Both isolates belonged to the internationally disseminated ST308/O11 high-risk lineage and exhibited resistance to all antimicrobial agents tested, consistent with a pandrug-resistant phenotype based on the antimicrobial panel evaluated. Whole-genome sequencing revealed a complex resistance architecture characterized by the presence of *blaNDM-1*, *blaOXA-10*, *blaOXA-488*, integron-associated multidrug resistance modules, resistance islands, chromosomal resistance mutations, and multiple efflux-associated systems.

In addition to antimicrobial resistance, both isolates carried a highly conserved repertoire of virulence-associated determinants, including the type III secretion system effector *exoU*, type VI secretion system components, iron acquisition systems, and biofilm-associated genes. The coexistence of extensive antimicrobial resistance and virulence-associated determinants within the same lineage highlights the clinical significance of ST308/O11 as an emerging high-risk clone in healthcare settings.

Comparative genomic analyses, including phylogenomic reconstruction, average nucleotide identity (ANI), pan-genome analysis, and genome-wide resistance and virulence profiling, demonstrated a high degree of conservation between the two Egyptian isolates and their placement within a globally distributed ST308 lineage. Despite this conserved genomic backbone, the Egyptian isolates possessed accessory resistance elements, including a *blaNDM-1*-associated resistance island, that were absent from their closest international relatives, emphasizing the ongoing contribution of horizontal gene transfer to the evolution of high-risk *P. aeruginosa* populations.

The novelty of this study lies in the integration of cohort-level screening of 100 VAP-associated clinical isolates with targeted whole-genome sequencing and comparative genomic analysis of the most extensively resistant isolates identified during surveillance. This approach enabled the characterization of a clinically important ST308/O11 lineage associated with fatal outcomes and provided region-specific genomic insights into the emergence of highly resistant *P. aeruginosa* in Egypt.

Overall, these findings underscore the value of genomic surveillance and integrated phenotypic–genotypic investigations for tracking the emergence, evolution, and dissemination of high-risk *P. aeruginosa* clones. Continued surveillance efforts incorporating larger genomic datasets and longitudinal monitoring will be essential for informing infection control strategies, antimicrobial stewardship programs, and public health interventions aimed at limiting the spread of extensively resistant lineages. 

## Supplementary Information

Supplementary Material 1. Supplementary Figure S1: Overview of the study design and analytical workflow. A total of 100 clinical samples obtained from ventilator-associated pneumonia (VAP) patients were screened using culture-based identification and antimicrobial susceptibility testing. Two Pseudomonas aeruginosa isolates exhibiting the most extensive resistance profiles were selected for whole-genome sequencing. Subsequent analyses included genome assembly and annotation, resistome and virulome profiling, mobile genetic element characterization, multilocus sequence typing (MLST), core-genome SNP phylogenetic reconstruction, pan-genome analysis, and integrated comparative genomic interpretation. Supplementary Figure S2A–B: Genome annotation overview and functional subsystem distribution of the two Pseudomonas aeruginosa isolates. (A) Circular genome map of Cairo2024_01 (SRR36023598) and (B) Cairo2025_02 (SRR36023597). From outer to inner rings, the maps display genomic coordinates, contigs/chromosomes, coding sequences (CDSs) on the forward strand, CDSs on the reverse strand, non-coding genomic features, antimicrobial resistance (AMR)-associated genes, virulence factor (VF)-associated genes, transporter-associated genes, predicted drug targets, GC content, and GC skew. Functional subsystem distributions are shown for both isolates. Overall, both genomes exhibited highly similar organization and annotation profiles, consistent with their close genomic relatedness. Supplementary Figure S3A–B: Comparative CARD-RGI resistome profiles of the two ST308 Pseudomonas aeruginosa isolates. (A) Cairo2024_01 (SRR36023598) and (B) Cairo2025_02 (SRR36023597). Sunburst plots illustrate the hierarchical classification of antimicrobial resistance determinants identified using CARD-RGI. Both isolates shared highly similar resistance gene repertoires, including carbapenemase (*blaNDM-1*), class D β-lactamases (*blaOXA-10* and *blaOXA-488*), aminoglycoside resistance determinants, fluoroquinolone resistance genes, sulfonamide resistance genes, and multiple efflux-associated components, supporting a highly conserved resistome architecture. Supplementary Figure S4A–B: Mobilome–antimicrobial resistance genomic context of the two Pseudomonas aeruginosa isolates. (A) Cairo2024_01 (SRR36023598) and (B) Cairo2025_02 (SRR36023597).

Supplementary Table S1. Antimicrobial susceptibility profiles and resistance classification of 100 clinical Pseudomonas aeruginosa isolates collected from Egyptian hospitals. Supplementary Table S2. Metadata for all genomes included in the phylogenomic analysis of Pseudomonas aeruginosa (52 genomes: two study isolates and 50 Mash-selected NCBI RefSeq references). Genome size and GC content were computed from the assembly sequences used in the analysis. Serogroup assignments are in silico O-antigen calls (Pasty). Isolation sources for reference genomes were obtained from NCBI BioSample records. Supplementary Table S3. Pairwise core-genome SNP distance matrix for all genomes included in the phylogenomic analysis. Supplementary Table S4 Variants of uncertain clinical significance. Supplementary Table S5 Complete genomic island annotations identified in Cairo2024_01 and Cairo2025_02. Supplementary Table S6. Mobile genetic elements associated with antimicrobial resistance regions in the Egyptian ST308 isolates. Supplementary Table S7. Selected virulence-associated genes identified in both Pseudomonas aeruginosa isolates (Cairo2024_01 and Cairo2025_02) based on VFDB analysis.

Supplementary Material 3.

## Data Availability

The datasets generated and analyzed during the current study are publicly available in the NCBI Sequence Read Archive (SRA). The two clinical isolates were deposited under accession numbers SRR36023598 (BioSample SAMN53246146; Cairo2024_01) and SRR36023597 (BioSample SAMN53246147; Cairo2025_02). Additional data supporting the findings of this study are included within the article and its supplementary materials.

## Abbreviations

ABRicate: Antibiotic Resistance and Virulence Gene Detection Tool
AF: Atrial Fibrillation
AMR: Antimicrobial Resistance
ARO: Antibiotic Resistance Ontology
AST: Antimicrobial Susceptibility Testing
BMD: Broth Microdilution
CARD: Comprehensive Antibiotic Resistance Database
CDS: Coding DNA Sequences
CGE: Center for Genomic Epidemiology
CLSI: Clinical and Laboratory Standards Institute
DM: Diabetes Mellitus
DNA: Deoxyribonucleic Acid
ETA: Endotracheal Aspirate
ESRD: End-Stage Renal Disease
GC: Guanine–Cytosine
HAC: High Accuracy (Basecalling Model)
HTN: Hypertension
ICU: Intensive Care Unit
IHD: Ischemic Heart Disease
IS: Insertion Sequence
LPS: Lipopolysaccharide
MALDI-TOF MS: Matrix-Assisted Laser Desorption/Ionization Time-of-Flight Mass Spectrometry
MDR: Multidrug-Resistant
MGE: Mobile Genetic Element
MIC: Minimum Inhibitory Concentration
MLST: Multilocus Sequence Typing
MHA: Mueller–Hinton Agar
ONT: Oxford Nanopore Technologies
OMPs: Outer Membrane Proteins
PATRIC: Pathosystems Resource Integration Center
PBS: Phosphate-Buffered Saline
ANI: Average Nucleotide Identity
PCR: Polymerase Chain Reaction
PDR: Pandrug-Resistant
PGFams: PATRIC Global Protein Families
RAST: Rapid Annotations using Subsystems Technology
RASTtk: RAST Toolkit
RGI: Resistance Gene Identifier
RND: Resistance–Nodulation–Division
rRNA: Ribosomal Ribonucleic Acid
SRA: Sequence Read Archive
ST: Sequence Type
T3SS: Type III Secretion System
T6SS: Type VI Secretion System
TSA: Tryptic Soy Agar
VAP: Ventilator-Associated Pneumonia
VFDB: Virulence Factors Database
WGS: Whole-Genome Sequencing
