# Supplementary material for: Comparative genomics of fatal ST308/O11 Pseudomonas aeruginosa from Egypt characterizes conserved resistance architecture, integron-associated multidrug resistance modules, and blaNDM-1 resistance islands
Source: BMC Microbiol. 2026 Aug 8;26:705. doi: 10.1186/s12866-026-05465-y (PMC13452102; doi:10.1186/s12866-026-05465-y)
Supplement: Supplementary file 1 [file 12866_2026_5465_MOESM3_ESM.pdf]

Ministry of Defense
Military Medical Academy
Health & Preventive Institute

The Institution Review Board of the Military Medical Academy (IRB -MMA) is constituted and operates according to institutional regulations and the national regulations. IRB is registered with the OHRP/FDA number (00014174 Military Medical Academy-Ministry of Defense IRB #1). (FWA 00034287). All activities related to human subjects' research is guided by the ethical principles of the Declaration of Helsinki and the Belmont report. IRB-MMA is in charge with review and approval of all researches involving human participants, animal research and lab experiments at MMA and other research sites. Approval in accordance with the Law Regulating Clinical Medical Research (214 of 2020) for a period of one calendar year.

Institutional Review Board Decision

On session dated 25/05/2024 The Institution Review Board of the Military Medical Academy discussed the study presented by: **Mohamed Gomaa Seadawy**

STUDY TITLE

Insights Into Antibiotic Resistance of Bacteria Through Advanced Genome Sequencing Technology

The IRB members met and reviewed the following presented documents for the above-named protocol

☒ Study protocol ☒ Arabic Consent ☒ Questionnaire

The attending members of military medical academy review board **APROVED** the study

Decision: **APPROVED**
Approval No: **17 -2025**

- *This approval is valid for **one year** only and has to be renewed if study extended.*
- *The patients have to be consented on the (Informed Consent Form) ICF stamped by IRB.*
- *The investigator(s) has to report any protocol changes or protocol deviation to the IRB.*

Signature

Major General Dr./ Mostafa Elnakib

IRB Chairman

Scanned with CamScanner
