# Supplementary material for: Comparative genomics of fatal ST308/O11 Pseudomonas aeruginosa from Egypt characterizes conserved resistance architecture, integron-associated multidrug resistance modules, and blaNDM-1 resistance islands
Source: BMC Microbiol. 2026 Aug 8;26:705. doi: 10.1186/s12866-026-05465-y (PMC13452102; doi:10.1186/s12866-026-05465-y)
Supplement: Supplementary file 2 [file 12866_2026_5465_MOESM2_ESM.docx]

### Supplementary Table S1.

**Antimicrobial susceptibility profiles and resistance classification of 100 clinical Pseudomonas aeruginosa isolates collected from Egyptian hospitals.**

| Sample ID | Isolate Code | Hospital | MEM | IPM | CAZ | FEP | CIP | LEV | TZP | COL | Profile |
| --- | --- | --- | --- | --- | --- | --- | --- | --- | --- | --- | --- |
| 1 | PA-01 | Kasr Al Ainy Hospital | R | R | R | S | R | S | R | S | MDR |
| 2 | PA-02 | Ain Shams University Hospitals | R | R | R | R | R | R | R | S | MDR |
| 3 | PA-03 | Alexandria Main University Hospital | R | R | S | S | R | S | R | S | MDR |
| 4 | PA-04 | Mansoura University Hospitals | R | R | R | R | R | R | R | S | MDR |
| 5 | PA-05 | Tanta University Hospital | S | S | S | S | S | S | S | S | Non-MDR |
| 6 | PA-06 | Zagazig University Hospitals | R | R | R | S | R | R | R | S | MDR |
| 7 | PA-07 | Assiut University Hospitals | R | R | S | S | S | R | R | S | MDR |
| 8 | PA-08 | Sohag University Hospital | R | R | R | R | S | R | R | S | MDR |
| 9 | PA-09 | Benha University Hospital | R | R | R | S | R | R | R | S | MDR |
| 10 | **SRR35813459** | **AlAzhar University Hospital** | **R** | **R** | **R** | **R** | **R** | **R** | **R** | **R** | **PDR** |
| 11 | **SRR35813460** | **Ain Shams University Hospital** | **R** | **R** | **R** | **R** | **R** | **R** | **R** | **R** | **PDR** |
| 12 | PA-12 | Beni-Suef University Hospital | R | R | R | S | S | R | R | S | MDR |
| 13 | PA-13 | Fayoum University Hospital | R | R | S | R | R | S | R | S | MDR |
| 14 | PA-14 | Helwan General Hospital | R | R | R | R | S | R | R | S | MDR |
| 15 | PA-15 | El Demerdash Hospital | R | R | R | R | R | R | R | S | MDR |
| 16 | PA-16 | Al-Azhar University Hospital | S | S | S | S | S | S | S | S | Non-MDR |
| 17 | PA-17 | Nasser Institute Hospital | R | R | S | R | R | R | R | S | MDR |
| 18 | PA-18 | National Cancer Institute Hospital | R | R | R | S | S | R | R | S | MDR |
| 19 | PA-19 | Dar Al Fouad Hospital | R | R | R | R | R | R | R | S | MDR |
| 20 | PA-20 | 57357 Hospital | R | R | R | S | S | R | R | S | MDR |
| 21 | PA-21 | Bahtim Central Hospital | R | R | R | S | S | R | R | S | MDR |
| 22 | PA-22 | Kafr El Sheikh General Hospital | R | R | R | R | S | R | R | S | MDR |
| 23 | PA-23 | Damietta General Hospital | R | R | R | S | R | R | R | S | MDR |
| 24 | PA-24 | Port Said General Hospital | R | R | R | S | R | R | R | S | MDR |
| 25 | PA-25 | El Mahalla Chest Hospital | R | R | R | R | R | R | R | S | MDR |
| 26 | PA-26 | Shebin El Kom Teaching Hospital | R | R | S | R | R | R | R | S | MDR |
| 27 | PA-27 | Damanhour Medical Institute | R | R | S | R | R | R | R | S | MDR |
| 28 | PA-28 | Matrouh General Hospital | R | R | S | S | S | R | R | S | MDR |
| 29 | PA-29 | Aswan University Hospital | R | R | R | R | S | R | R | S | MDR |
| 30 | PA-30 | Qena General Hospital | R | R | R | S | R | S | R | S | MDR |
| 31 | PA-31 | Luxor International Hospital | R | R | R | R | R | S | R | S | MDR |
| 32 | PA-32 | Hurghada General Hospital | R | R | R | R | R | S | R | S | MDR |
| 33 | PA-33 | Sharm El Sheikh International Hospital | R | R | R | R | S | R | R | S | MDR |
| 34 | PA-34 | Arish General Hospital | R | R | R | S | S | R | R | S | MDR |
| 35 | PA-35 | Ismailia Chest Hospital | R | R | R | S | R | R | R | S | MDR |
| 36 | PA-36 | Suez General Hospital | R | R | R | R | S | R | R | S | MDR |
| 37 | PA-37 | New Cairo Hospital | R | R | R | R | R | R | R | S | MDR |
| 38 | PA-38 | 6th of October Central Hospital | R | R | R | R | R | R | R | S | MDR |
| 39 | PA-39 | Imbaba General Hospital | R | R | R | R | R | R | R | S | MDR |
| 40 | PA-40 | Banha Teaching Hospital | R | R | R | R | R | R | R | S | MDR |
| 41 | PA-41 | Kafr El Dawar General Hospital | R | R | S | R | S | R | R | S | MDR |
| 42 | PA-42 | Abbasia Fever Hospital | R | R | R | R | R | R | R | S | MDR |
| 43 | PA-43 | Abbassia Chest Hospital | R | R | R | R | R | R | R | S | MDR |
| 44 | PA-44 | Mabarra Hospital | R | R | R | S | S | R | R | S | MDR |
| 45 | PA-45 | Ras El Teen Hospital | S | S | S | S | S | S | S | S | Non-MDR |
| 46 | PA-46 | Gamal Abdel Nasser Hospital | R | R | R | R | R | R | R | S | MDR |
| 47 | PA-47 | Koum Hamada Central Hospital | R | R | R | S | S | R | R | S | MDR |
| 48 | PA-48 | Itay El Baroud Hospital | R | R | R | R | R | R | R | S | MDR |
| 49 | PA-49 | Mit Ghamr General Hospital | S | S | S | S | S | S | S | S | Non-MDR |
| 50 | PA-50 | Talkha Central Hospital | S | S | S | S | S | S | S | S | Non-MDR |
| 51 | PA-51 | Aga Central Hospital | R | R | R | R | S | R | R | S | MDR |
| 52 | PA-52 | Belqas General Hospital | R | R | R | R | R | R | R | S | MDR |
| 53 | PA-53 | Kafr Saqr Hospital | R | R | R | R | R | R | R | S | MDR |
| 54 | PA-54 | Abu Hammad Hospital | R | R | R | S | R | R | R | S | MDR |
| 55 | PA-55 | Faqous General Hospital | R | R | R | R | S | R | R | S | MDR |
| 56 | PA-56 | Desouk General Hospital | R | R | R | R | R | R | R | S | MDR |
| 57 | PA-57 | Baltim Central Hospital | R | R | R | R | R | R | R | S | MDR |
| 58 | PA-58 | Rosetta General Hospital | R | R | R | R | R | R | R | S | MDR |
| 59 | PA-59 | Edfina Central Hospital | R | R | R | S | R | R | R | S | MDR |
| 60 | PA-60 | Kom Ombo Central Hospital | R | R | R | R | R | R | R | S | MDR |
| 61 | PA-61 | Edfu General Hospital | R | R | R | R | R | R | R | S | MDR |
| 62 | PA-62 | Nag Hammadi Hospital | R | R | R | R | R | R | R | S | MDR |
| 63 | PA-63 | Qus Central Hospital | R | R | R | R | R | R | R | S | MDR |
| 64 | PA-64 | Esna Central Hospital | R | R | R | R | R | R | R | S | MDR |
| 65 | PA-65 | Armant Hospital | R | R | R | R | R | R | R | S | MDR |
| 66 | PA-66 | Mallawi General Hospital | R | R | R | R | R | R | R | S | MDR |
| 67 | PA-67 | Maghagha Central Hospital | R | R | R | S | S | R | R | S | MDR |
| 68 | PA-68 | Samalut General Hospital | R | R | R | R | R | R | R | S | MDR |
| 69 | PA-69 | El Qusiya Central Hospital | R | R | R | R | R | R | R | S | MDR |
| 70 | PA-70 | Abnoub Central Hospital | R | R | R | S | S | R | R | S | MDR |
| 71 | PA-71 | Tahta General Hospital | R | R | R | R | R | R | R | S | MDR |
| 72 | PA-72 | Girga General Hospital | R | R | R | S | S | R | R | S | MDR |
| 73 | PA-73 | Akhmim Central Hospital | R | R | R | R | R | R | R | S | MDR |
| 74 | PA-74 | Basyoun Central Hospital | R | R | R | R | R | R | R | S | MDR |
| 75 | PA-75 | Kafr El Zayat General Hospital | R | R | R | R | R | R | R | S | MDR |
| 76 | PA-76 | Zefta General Hospital | R | R | R | S | R | S | R | S | MDR |
| 77 | PA-77 | Ashmoun General Hospital | R | R | R | R | R | R | R | S | MDR |
| 78 | PA-78 | Menouf General Hospital | R | R | R | R | R | R | R | S | MDR |
| 79 | PA-79 | Quesna Central Hospital | R | R | R | R | R | R | R | S | MDR |
| 80 | PA-80 | El Santa Central Hospital | R | R | R | R | R | R | R | S | MDR |
| 81 | PA-81 | Shibin El Qanater Hospital | R | R | R | R | R | R | R | S | MDR |
| 82 | PA-82 | Toukh Central Hospital | R | R | R | R | R | R | R | S | MDR |
| 83 | PA-83 | Khanka Central Hospital | R | R | R | S | R | S | R | S | MDR |
| 84 | PA-84 | El Bagour General Hospital | R | R | R | R | R | R | R | S | MDR |
| 85 | PA-85 | Sadat General Hospital | S | S | S | S | S | S | S | S | Non-MDR |
| 86 | PA-86 | Matay Central Hospital | R | R | R | R | R | R | R | S | MDR |
| 87 | PA-87 | Dayrout Central Hospital | R | R | R | S | R | R | R | S | MDR |
| 88 | PA-88 | El Badari Hospital | R | R | S | S | R | R | R | S | MDR |
| 89 | PA-89 | Farshout Central Hospital | R | R | R | R | R | R | R | S | MDR |
| 90 | PA-90 | Qift Central Hospital | R | R | S | R | S | R | R | S | MDR |
| 91 | PA-91 | Marsa Matrouh Fever Hospital | R | R | R | R | R | R | R | S | MDR |
| 92 | PA-92 | Siwa Central Hospital | R | R | S | R | R | R | R | S | MDR |
| 93 | PA-93 | El Tor General Hospital | R | R | R | S | R | R | R | S | MDR |
| 94 | PA-94 | Dahab Central Hospital | R | R | R | S | R | R | R | S | MDR |
| 95 | PA-95 | Nuweiba Hospital | R | R | R | R | R | R | R | S | MDR |
| 96 | PA-96 | Bir El Abd Hospital | R | R | R | S | S | R | R | S | MDR |
| 97 | PA-97 | Sheikh Zuweid Hospital | R | R | R | R | R | R | R | S | MDR |
| 98 | PA-98 | Rafah Central Hospital | R | R | R | S | R | R | R | S | MDR |
| 99 | PA-99 | Heliopolis Hospital | R | R | R | R | R | R | R | S | MDR |
| 100 | PA-100 | Cairo Specialized Hospital | R | R | S | R | S | R | R | S | MDR |

**Supplementary Table S2**

**Metadata for all genomes included in the phylogenomic analysis of Pseudomonas aeruginosa (52 genomes: two study isolates and 50 Mash-selected NCBI RefSeq references). Genome size and GC content were computed from the assembly sequences used in the analysis. Serogroup assignments are in silico O-antigen calls (Pasty). Isolation sources for reference genomes were obtained from NCBI BioSample records.**

| Genome ID | Accession number | Country | Isolation source | Sequence type (ST) | Serogroup | Genome size (bp) | GC content (%) | Reference/source |
| --- | --- | --- | --- | --- | --- | --- | --- | --- |
| Cairo2024_01 | **SRR36023598** | **Egypt** | **Endotracheal aspirate; ventilator-associated pneumonia (ICU patient)** | **308** | **O11** | **6,764,468** | **66.15** | **This study (Oxford Nanopore WGS; draft assembly)** |
| Cairo2025_02 | **SRR36023597** | **Egypt** | **Endotracheal aspirate; ventilator-associated pneumonia (ICU patient)** | **308** | **O11** | **6,995,336** | **65.96** | **This study (Oxford Nanopore WGS; draft assembly)** |
| 01_GCF_056834515.1 | GCF_056834515.1 | Argentina: Ciudad Autonoma de Buenos Aires | blood | 308 | O11 | 6,910,006 | 66.01 | NCBI RefSeq (Mash-closest comparative panel) |
| 02_GCF_052695645.1 | GCF_052695645.1 | Canada: Calgary | sputum | 308 | O11 | 6,954,734 | 66.04 | NCBI RefSeq (Mash-closest comparative panel) |
| 03_GCF_054958065.1 | GCF_054958065.1 | China: Sichuan | bronchoalveolar lavage fluid | 5159 | O11 | 6,425,849 | 66.44 | NCBI RefSeq (Mash-closest comparative panel) |
| 04_GCF_055432725.1 | GCF_055432725.1 | Australia:Perth | Respiratory culture | 775 | O3 | 6,350,265 | 66.46 | NCBI RefSeq (Mash-closest comparative panel) |
| 05_GCF_051416385.1 | GCF_051416385.1 | China: Hangzhou | clinical | 1076 | O11 | 7,138,065 | 65.50 | NCBI RefSeq (Mash-closest comparative panel) |
| 06_GCF_054126015.1 | GCF_054126015.1 | Australia:Perth | Urine culture | 2555 | O11 | 6,453,556 | 66.43 | NCBI RefSeq (Mash-closest comparative panel) |
| 07_GCF_051015985.1 | GCF_051015985.1 | Denmark | ear canal swab | 1284 | O11 | 6,612,504 | 66.21 | NCBI RefSeq (Mash-closest comparative panel) |
| 08_GCF_052377975.1 | GCF_052377975.1 | China: Jiangsu Province | Urine | 2555 | O11 | 6,522,294 | 66.37 | NCBI RefSeq (Mash-closest comparative panel) |
| 09_GCF_052327275.1 | GCF_052327275.1 | Thailand: Phayao | urine | 357 | O11 | 6,780,605 | 65.96 | NCBI RefSeq (Mash-closest comparative panel) |
| 10_GCF_054130155.1 | GCF_054130155.1 | Australia:Perth | Blood culture | 207 | O1 | 6,552,820 | 66.24 | NCBI RefSeq (Mash-closest comparative panel) |
| 11_GCF_057592735.1 | GCF_057592735.1 | Belgium | Respiratory sample | 207 | O1 | 6,502,764 | 66.23 | NCBI RefSeq (Mash-closest comparative panel) |
| 12_GCF_057592755.1 | GCF_057592755.1 | Belgium | Respiratory sample | 207 | O1 | 6,502,673 | 66.23 | NCBI RefSeq (Mash-closest comparative panel) |
| 13_GCF_057592775.1 | GCF_057592775.1 | Belgium | Respiratory sample | 207 | O1 | 6,502,677 | 66.23 | NCBI RefSeq (Mash-closest comparative panel) |
| 14_GCF_054130275.1 | GCF_054130275.1 | Australia:Melbourne | Respiratory culture | 2620 | O6 | 6,465,469 | 66.37 | NCBI RefSeq (Mash-closest comparative panel) |
| 15_GCF_053291805.1 | GCF_053291805.1 | South Africa | Tomatoes (point of harvest) | 313 | Not typed | 6,265,348 | 64.36 | NCBI RefSeq (Mash-closest comparative panel) |
| 16_GCF_052327285.1 | GCF_052327285.1 | Thailand: Samut Songkhram | urine | Not typed | O11 | 6,866,642 | 65.85 | NCBI RefSeq (Mash-closest comparative panel) |
| 17_GCF_053320215.1 | GCF_053320215.1 | India: Pune | soil | 313 | O1 | 6,494,396 | 66.27 | NCBI RefSeq (Mash-closest comparative panel) |
| 18_GCF_050908025.1 | GCF_050908025.1 | Taiwan: Taichung | sputum | 207 | O1 | 6,669,166 | 66.12 | NCBI RefSeq (Mash-closest comparative panel) |
| 19_GCF_053360585.1 | GCF_053360585.1 | Taiwan | not applicable | 298 | O11 | 6,959,156 | 66.01 | NCBI RefSeq (Mash-closest comparative panel) |
| 20_GCF_052328305.1 | GCF_052328305.1 | Thailand: Nakhon Sawan | urine | Not typed | O11 | 6,876,250 | 65.88 | NCBI RefSeq (Mash-closest comparative panel) |
| 21_GCF_057810675.1 | GCF_057810675.1 | Germany:Hamburg | Host: Homo sapiens | 773 | O11 | 6,834,870 | 65.97 | NCBI RefSeq (Mash-closest comparative panel) |
| 22_GCF_057961515.1 | GCF_057961515.1 | USA: Charlottesville, Virginia | sputum | 773 | O11 | 6,862,507 | 66.12 | NCBI RefSeq (Mash-closest comparative panel) |
| 23_GCF_051257825.1 | GCF_051257825.1 | USA | Tissue | 1047 | O7 | 6,883,718 | 66.04 | NCBI RefSeq (Mash-closest comparative panel) |
| 24_GCF_053360575.1 | GCF_053360575.1 | Taiwan | Tissue | 235 | O11 | 6,939,997 | 66.10 | NCBI RefSeq (Mash-closest comparative panel) |
| 25_GCF_053360625.1 | GCF_053360625.1 | Taiwan | Tissue | 298 | O11 | 6,909,566 | 65.98 | NCBI RefSeq (Mash-closest comparative panel) |
| 26_GCF_057811355.1 | GCF_057811355.1 | Germany:Hamburg | Host: Homo sapiens | 235 | O11 | 6,764,342 | 65.94 | NCBI RefSeq (Mash-closest comparative panel) |
| 27_GCF_052329215.1 | GCF_052329215.1 | Thailand: Bangkok | urine | Not typed | O11 | 6,962,035 | 65.75 | NCBI RefSeq (Mash-closest comparative panel) |
| 28_GCF_051257855.1 | GCF_051257855.1 | USA | Urine | 1047 | O7 | 6,945,820 | 66.02 | NCBI RefSeq (Mash-closest comparative panel) |
| 29_GCF_056669625.1 | GCF_056669625.1 | not collected | Host: not collected | 377 | O7 | 6,764,196 | 66.16 | NCBI RefSeq (Mash-closest comparative panel) |
| 30_GCF_051122945.1 | GCF_051122945.1 | Taiwan: Taichung | Pus | 207 | O1 | 6,746,654 | 66.05 | NCBI RefSeq (Mash-closest comparative panel) |
| 31_GCF_050858815.1 | GCF_050858815.1 | Taiwan: Taichung | urine | Not typed | O11 | 6,780,815 | 66.26 | NCBI RefSeq (Mash-closest comparative panel) |
| 32_GCF_051122965.1 | GCF_051122965.1 | Taiwan: Taichung | sputum | Not typed | O11 | 6,804,130 | 66.28 | NCBI RefSeq (Mash-closest comparative panel) |
| 33_GCF_052975055.1 | GCF_052975055.1 | China: Hangzhou | Host: Homo sapiens | 207 | O1 | 7,174,840 | 65.56 | NCBI RefSeq (Mash-closest comparative panel) |
| 34_GCF_051015935.1 | GCF_051015935.1 | United Kingdom | ear canal swab | 815 | O11 | 6,846,060 | 66.11 | NCBI RefSeq (Mash-closest comparative panel) |
| 35_GCF_053372525.1 | GCF_053372525.1 | Taiwan | not applicable | 207 | O1 | 7,200,915 | 65.47 | NCBI RefSeq (Mash-closest comparative panel) |
| 36_GCF_051353645.1 | GCF_051353645.1 | China: Xi'an | Endotracheal intubation | 357 | O11 | 7,375,339 | 65.46 | NCBI RefSeq (Mash-closest comparative panel) |
| 37_GCF_050908045.1 | GCF_050908045.1 | Taiwan: Taichung | sputum | Not typed | O11 | 6,818,143 | 66.26 | NCBI RefSeq (Mash-closest comparative panel) |
| 38_GCF_051122825.1 | GCF_051122825.1 | Taiwan: Taichung | blood | 207 | O1 | 6,625,024 | 66.16 | NCBI RefSeq (Mash-closest comparative panel) |
| 39_GCF_053360725.1 | GCF_053360725.1 | Taiwan | Urine | 253 | O10 | 6,805,634 | 66.03 | NCBI RefSeq (Mash-closest comparative panel) |
| 40_GCF_057810915.1 | GCF_057810915.1 | Germany:Hamburg | Host: Homo sapiens | 357 | O11 | 6,927,002 | 65.62 | NCBI RefSeq (Mash-closest comparative panel) |
| 41_GCF_052329085.1 | GCF_052329085.1 | Thailand: Nakhon Sawan | urine | 357 | O11 | 7,021,992 | 65.77 | NCBI RefSeq (Mash-closest comparative panel) |
| 42_GCF_051257835.1 | GCF_051257835.1 | Ukraine | environmental | 1047 | O7 | 6,984,853 | 65.82 | NCBI RefSeq (Mash-closest comparative panel) |
| 43_GCF_052327325.1 | GCF_052327325.1 | Thailand: Chonburi | urine | 357 | O11 | 6,887,135 | 66.05 | NCBI RefSeq (Mash-closest comparative panel) |
| 44_GCF_057811375.1 | GCF_057811375.1 | Germany:Hamburg | Host: Homo sapiens | 235 | O11 | 6,883,898 | 65.82 | NCBI RefSeq (Mash-closest comparative panel) |
| 45_GCF_050908115.1 | GCF_050908115.1 | Taiwan: Taichung | urine | Not typed | O11 | 6,847,459 | 66.19 | NCBI RefSeq (Mash-closest comparative panel) |
| 46_GCF_005048525.2 | GCF_005048525.2 | USA:Boston | bronchoalveolar lavage | 235 | O11 | 6,868,833 | 66.02 | NCBI RefSeq (Mash-closest comparative panel) |
| 47_GCF_056858575.1 | GCF_056858575.1 | China: Hangzhou | clinical | 235 | O11 | 6,903,817 | 66.01 | NCBI RefSeq (Mash-closest comparative panel) |
| 48_GCF_054129955.1 | GCF_054129955.1 | Australia:Perth | Wound culture | 313 | O1 | 6,596,646 | 66.20 | NCBI RefSeq (Mash-closest comparative panel) |
| 49_GCF_051905005.1 | GCF_051905005.1 | Viet Nam: Hanoi | Sputum specimen | 1971 | O11 | 6,589,538 | 66.17 | NCBI RefSeq (Mash-closest comparative panel) |
| 50_GCF_056324585.1 | GCF_056324585.1 | South Korea | Host: Homo sapiens | 773 | O11 | 6,986,164 | 66.01 | NCBI RefSeq (Mash-closest comparative panel) |

**Supplementary Table S3.**

**Pairwise core-genome SNP distance matrix for all genomes included in the phylogenomic analysis.**

**Dataset summary**

| Parameter | Value |
| --- | --- |
| Total genomes analyzed | 52 |
| Panaroo core genes (≥99% presence) | 5,085 |
| Core genes in filtered alignment | 4,998 |
| SNP sites used in phylogeny (snp-sites -m) | 144,617 |
| Cairo2024_01 ↔ Cairo2025_02 | 74 SNPs |

**Distances to study isolates:-**

**All genomes ranked by core SNP distance to Cairo2024_01. Study isolates are highlighted.**

| Genome ID | Country | SNPs vs Cairo2024_01 | SNPs vs Cairo2025_02 |
| --- | --- | --- | --- |
| Cairo2024_01 | **Egypt** | **0** | **74** |
| Cairo2025_02 | **Egypt** | **74** | **0** |
| 01_GCF_056834515.1 | Argentina: Ciudad Autonoma de Buenos Aires | 137 | 155 |
| 02_GCF_052695645.1 | Canada: Calgary | 258 | 276 |
| 49_GCF_051905005.1 | Viet Nam: Hanoi | 29,457 | 29,483 |
| 50_GCF_056324585.1 | South Korea | 29,616 | 29,643 |
| 22_GCF_057961515.1 | USA: Charlottesville, Virginia | 29,624 | 29,674 |
| 21_GCF_057810675.1 | Germany:Hamburg | 30,083 | 30,111 |
| 05_GCF_051416385.1 | China: Hangzhou | 30,746 | 30,756 |
| 46_GCF_005048525.2 | USA:Boston | 30,989 | 31,013 |
| 09_GCF_052327275.1 | Thailand: Phayao | 31,022 | 31,045 |
| 43_GCF_052327325.1 | Thailand: Chonburi | 31,063 | 31,087 |
| 07_GCF_051015985.1 | Denmark | 31,070 | 31,093 |
| 29_GCF_056669625.1 | not collected | 31,073 | 31,097 |
| 16_GCF_052327285.1 | Thailand: Samut Songkhram | 31,165 | 31,188 |
| 23_GCF_051257825.1 | USA | 31,170 | 31,151 |
| 31_GCF_050858815.1 | Taiwan: Taichung | 31,186 | 31,209 |
| 45_GCF_050908115.1 | Taiwan: Taichung | 31,187 | 31,210 |
| 32_GCF_051122965.1 | Taiwan: Taichung | 31,188 | 31,211 |
| 41_GCF_052329085.1 | Thailand: Nakhon Sawan | 31,194 | 31,217 |
| 37_GCF_050908045.1 | Taiwan: Taichung | 31,199 | 31,222 |
| 24_GCF_053360575.1 | Taiwan | 31,201 | 31,224 |
| 47_GCF_056858575.1 | China: Hangzhou | 31,225 | 31,247 |
| 28_GCF_051257855.1 | USA | 31,242 | 31,223 |
| 42_GCF_051257835.1 | Ukraine | 31,247 | 31,228 |
| 20_GCF_052328305.1 | Thailand: Nakhon Sawan | 31,275 | 31,299 |
| 36_GCF_051353645.1 | China: Xi'an | 31,282 | 31,306 |
| 27_GCF_052329215.1 | Thailand: Bangkok | 31,294 | 31,317 |
| 40_GCF_057810915.1 | Germany:Hamburg | 31,737 | 31,759 |
| 26_GCF_057811355.1 | Germany:Hamburg | 32,000 | 32,023 |
| 44_GCF_057811375.1 | Germany:Hamburg | 32,181 | 32,204 |
| 15_GCF_053291805.1 | South Africa | 32,486 | 32,521 |
| 33_GCF_052975055.1 | China: Hangzhou | 32,663 | 32,698 |
| 17_GCF_053320215.1 | India: Pune | 33,313 | 33,347 |
| 19_GCF_053360585.1 | Taiwan | 33,569 | 33,583 |
| 34_GCF_051015935.1 | United Kingdom | 33,572 | 33,585 |
| 25_GCF_053360625.1 | Taiwan | 33,618 | 33,634 |
| 03_GCF_054958065.1 | China: Sichuan | 34,065 | 34,076 |
| 08_GCF_052377975.1 | China: Jiangsu Province | 34,423 | 34,439 |
| 06_GCF_054126015.1 | Australia:Perth | 34,461 | 34,478 |
| 14_GCF_054130275.1 | Australia:Melbourne | 34,648 | 34,657 |
| 11_GCF_057592735.1 | Belgium | 34,980 | 35,015 |
| 13_GCF_057592775.1 | Belgium | 34,982 | 35,017 |
| 12_GCF_057592755.1 | Belgium | 34,982 | 35,017 |
| 38_GCF_051122825.1 | Taiwan: Taichung | 34,984 | 35,019 |
| 18_GCF_050908025.1 | Taiwan: Taichung | 34,984 | 35,019 |
| 35_GCF_053372525.1 | Taiwan | 34,994 | 35,029 |
| 30_GCF_051122945.1 | Taiwan: Taichung | 34,998 | 35,033 |
| 10_GCF_054130155.1 | Australia:Perth | 35,007 | 35,042 |
| 48_GCF_054129955.1 | Australia:Perth | 35,551 | 35,584 |
| 39_GCF_053360725.1 | Taiwan | 37,126 | 37,138 |
| 04_GCF_055432725.1 | Australia:Perth | 52,624 | 52,660 |

**Closest reference genomes to each study isolate:**

| Study isolate | Rank | Reference genome | Core SNP distance |
| --- | --- | --- | --- |
| Cairo2024_01 | 1 | 01_GCF_056834515.1 (Argentina: Ciudad Autonoma de Buenos Aires) | 137 |
|  | 2 | 02_GCF_052695645.1 (Canada: Calgary) | 258 |
|  | 3 | 49_GCF_051905005.1 (Viet Nam: Hanoi) | 29,457 |
|  | 4 | 50_GCF_056324585.1 (South Korea) | 29,616 |
|  | 5 | 22_GCF_057961515.1 (USA: Charlottesville, Virginia) | 29,624 |
| Cairo2025_02 | 1 | 01_GCF_056834515.1 (Argentina: Ciudad Autonoma de Buenos Aires) | 155 |
|  | 2 | 02_GCF_052695645.1 (Canada: Calgary) | 276 |
|  | 3 | 49_GCF_051905005.1 (Viet Nam: Hanoi) | 29,483 |
|  | 4 | 50_GCF_056324585.1 (South Korea) | 29,643 |
|  | 5 | 22_GCF_057961515.1 (USA: Charlottesville, Virginia) | 29,674 |

**ST308 clade verification:**

1. ST typing and serogroup: Both closest reference genomes (01_GCF_056834515.1, Argentina: Ciudad Autonoma de Buenos Aires; 02_GCF_052695645.1, Canada: Calgary) were assigned ST308 and serogroup O11, matching both study isolates. These four genomes were the only ST308 representatives in the 52-genome panel.
2. All four genomes were present in the final core_gene_alignment_snps.aln (144,617 SNP columns) and in the IQ-TREE SNP phylogeny (GTR+ASC; bootstrap = 2,000).
3. Phylogenetic concordance: In the final SNP tree the four genomes formed a supported monophyletic clade (Cairo2024_01, Cairo2025_02), Argentina ST308, Canada ST308).
4. Distance gap interpretation: Core SNP distances within the ST308 clade ranged from 74 to 299 SNPs. The next closest reference (49_GCF_051905005.1; ST1971) was 29,457 SNPs from Cairo2024_01 (+29,199 SNPs beyond the Canadian comparator). This discontinuity reflects lineage separation, not a selection error: Mash/ANI remain high (~99.9%) across diverse *P. aeruginosa* references, whereas core SNP analysis resolves clone membership.

| Genome | ST / serogroup | SNPs vs Cairo2024_01 | Notes |
| --- | --- | --- | --- |
| Cairo2024_01 | ST308 / O11 | 0 | Study isolate |
| Cairo2025_02 | ST308 / O11 | 74 | Study isolate; nearest neighbour |
| 01_GCF_056834515.1 | ST308 / O11 | 137 | Closest reference; Mash rank 1 |
| 02_GCF_052695645.1 | ST308 / O11 | 258 | Second closest reference; Mash rank 2 |

#### Supplementary Table S4

#### Variants of uncertain clinical significance

| Gene | Variant | Interpretation |
| --- | --- | --- |
| *parE* | D533E | Outside established QRDR |
| *parC* | P752T | Outside QRDR hotspot |
| *nalC* | D79E | Unknown significance |
| *nalD* | V96M | Unknown significance |
| *mexZ* | G89S | Possible regulator alteration |
| *ampR* | A114E | Possible regulatory effect |
| *oprD* | A315G | Non-curated polymorphism |
| *oprD* | F170L | Non-curated polymorphism |
| *oprD* | K115T | Non-curated polymorphism |
| *oprD* | R310E | Non-curated polymorphism |
| *oprD* | T103S | Non-curated polymorphism |
| *oprD* | V189T | Non-curated polymorphism |
| *oprD* | EP185QG | Non-curated polymorphism |
| *pmrB* | A4T | Non-curated polymorphism |
| *pmrB* | G68S | Non-curated polymorphism |
| *pmrB* | S2P | Non-curated polymorphism |
| *pmrB* | Y345H | Non-curated polymorphism |

**Supplementary Table S5.**

**Complete genomic island annotations identified in Cairo2024_01 and Cairo2025_02.**

| Island ID | Island type | Cairo2024_01 region | Cairo2025_02 region | Major genetic content |
| --- | --- | --- | --- | --- |
| GI_14 | Resistance island | 1.89–1.93 Mb | Corresponding region | *sul2*, *floR*, Tn5393, *aph(3'')-Ib*, mercury operon |
| GI_26 | Resistance island | 3.89–3.93 Mb | Corresponding region | *aac(6')-II*, *dfrB5*, *aac(3)-Id*, *qnrVC1*, *aadA11* |
| GI_27 | Resistance island | 4.31–4.43 Mb | Corresponding region | *sul1*, *rmtF*, *blaOXA-10*, *blaPAC-1*, *qacEΔ1* |
| GI_32 | Resistance–virulence island | Corresponding region | Corresponding region | *exoU*-containing genomic island |
| GI_42 | Resistance island | 6.61–6.64 Mb | Corresponding region | *blaNDM-1*, *ble*, *msrE*, ISVsa3 |
| Additional islands | Virulence-associated islands | Multiple regions | Multiple regions | T3SS, T6SS, iron acquisition, adhesion, and accessory genomic content |

**Summary of genomic island content.**

| Island category | Cairo2024_01 | Cairo2025_02 |
| --- | --- | --- |
| Resistance islands | 5 | 6 |
| Resistance–virulence islands | 9 | 8 |
| Virulence-associated islands | 23 | 20 |
| Additional genomic islands | 5 | 6 |

### Supplementary Table S6.

### Mobile genetic elements associated with antimicrobial resistance regions in the Egyptian ST308 isolates.

| Resistance region | Associated MGE | Family / Type | Putative role |
| --- | --- | --- | --- |
| *qnrVC1* cassette array | XerC recombinase | Site-specific recombinase | Recombination and cassette mobility |
| *sul1-rmtF-blaOXA-10* module | TnAs1 | Tn3-family transposon | Resistance region mobilization |
| *blaNDM-1* resistance island | ISVsa3 | IS91-family insertion sequence | Association with resistance island structure |
| *floR-sul2* resistance island | Tn5393 | Transposon | Mobilization of phenicol and sulfonamide resistance genes |

**Supplementary Table S7.**

**Selected virulence-associated genes identified in both *Pseudomonas aeruginosa* isolates (Cairo2024_01 and Cairo2025_02) based on VFDB analysis.**

| Gene | % Coverage | % Identity | Function |
| --- | --- | --- | --- |
| *pvdA* | 100 | 83.56 | Pyoverdine biosynthesis enzyme involved in iron acquisition |
| *pvdS* | 100 | 98.05 | Sigma factor regulating iron acquisition systems |
| *algD* | 100 | 99.69 | Alginate biosynthesis enzyme associated with biofilm formation |
| *algU* | 100 | 99.14 | Sigma factor regulating stress response and alginate production |
| *algR* | 100 | 99.60 | Regulator of alginate production and virulence gene expression |
| *mucA* | 100 | 99.14 | Negative regulator of alginate production |
| *mucB* | 100 | 99.05 | Modulator of alginate regulatory pathway |
| *pilB* | 100 | 88.42 | Type IV pilus assembly ATPase |
| *pilY1* | 99.43 | 94.21 | Pilus-associated adhesion protein |
| *pilR* | 100 | 99.33 | Regulator of pilus biogenesis |
| *exoT* | 100 | 99.64 | Type III secretion system effector |
| *exoU* | 100 | 99.95 | Type III secretion system effector associated with cytotoxicity |
| *vgrG1a* | 100 | 99.59 | Type VI secretion system component |
| *hsiJ1* | 100 | 99.70 | T6SS structural protein |
| *dotU1* | 100 | 99.78 | T6SS membrane-associated protein |
| *icmF1* | 100 | 98.82 | Core T6SS structural protein |
| *plcH* | 100 | 99.59 | Phospholipase involved in host interaction |
| *phzM* | 100 | 99.10 | Phenazine biosynthesis enzyme |
| *phzS* | 100 | 98.51 | Phenazine biosynthesis enzyme |
| *phzH* | 100 | 99.34 | Phenazine-modifying enzyme |
