# Supplementary material for: Comparative genomics of fatal ST308/O11 Pseudomonas aeruginosa from Egypt characterizes conserved resistance architecture, integron-associated multidrug resistance modules, and blaNDM-1 resistance islands
Source: BMC Microbiol. 2026 Aug 8;26:705. doi: 10.1186/s12866-026-05465-y (PMC13452102; doi:10.1186/s12866-026-05465-y)

Supplementary Figure S1

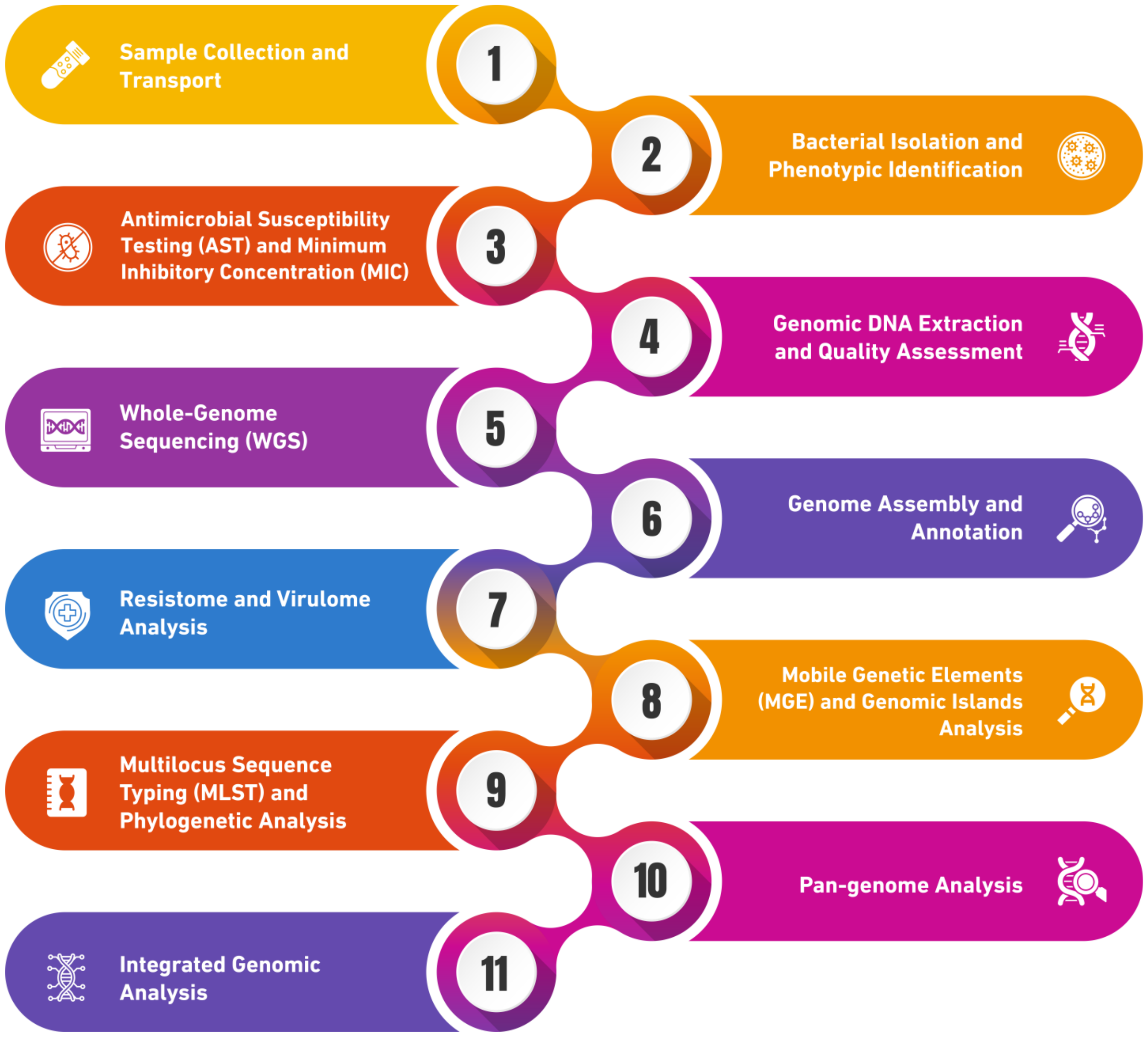

Supplementary Figure S2A

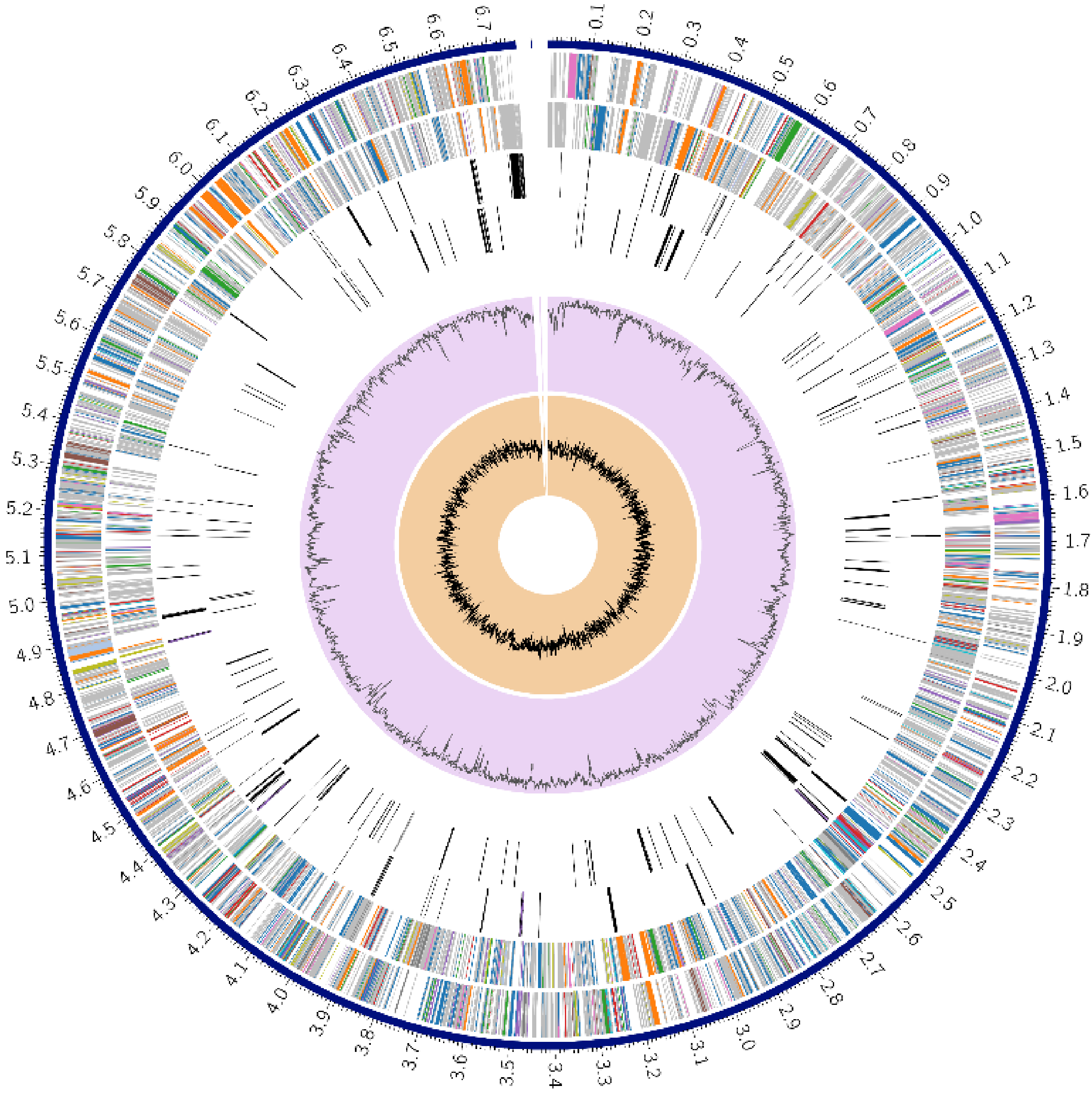

Supplementary Figure S2B

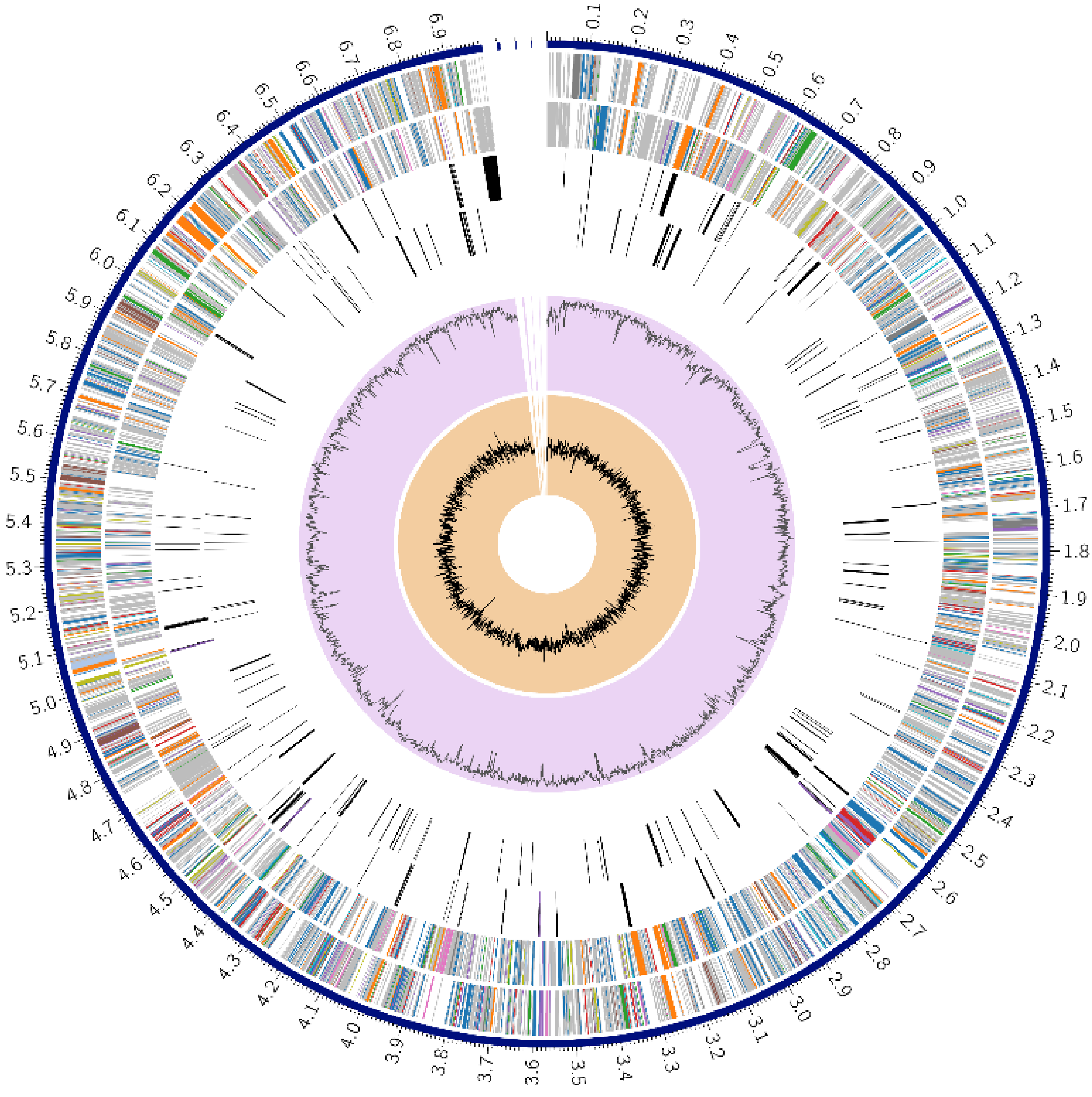

Supplementary Figure S3A

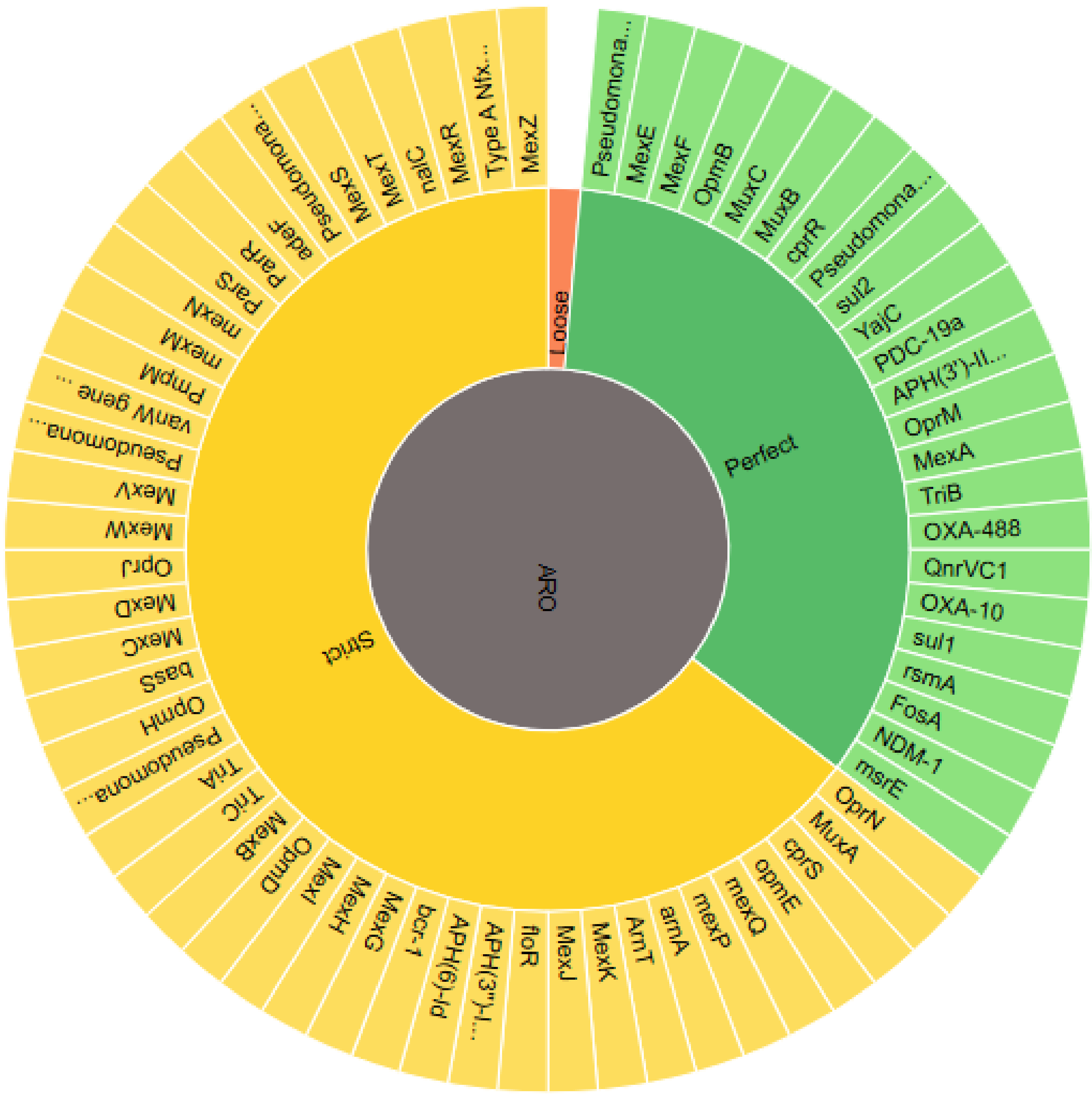

Supplementary Figure S3B

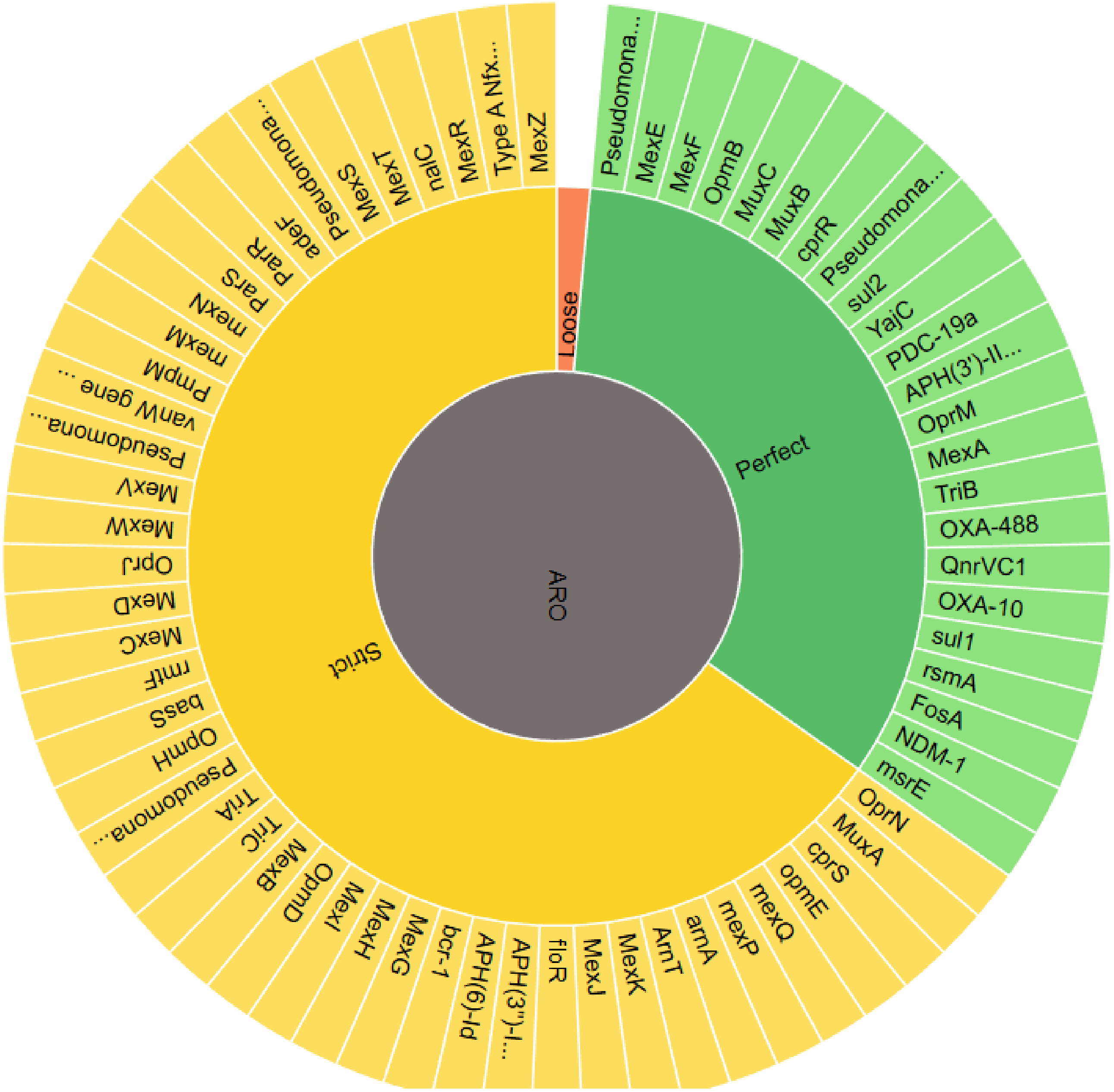

Supplementary Figure S4A

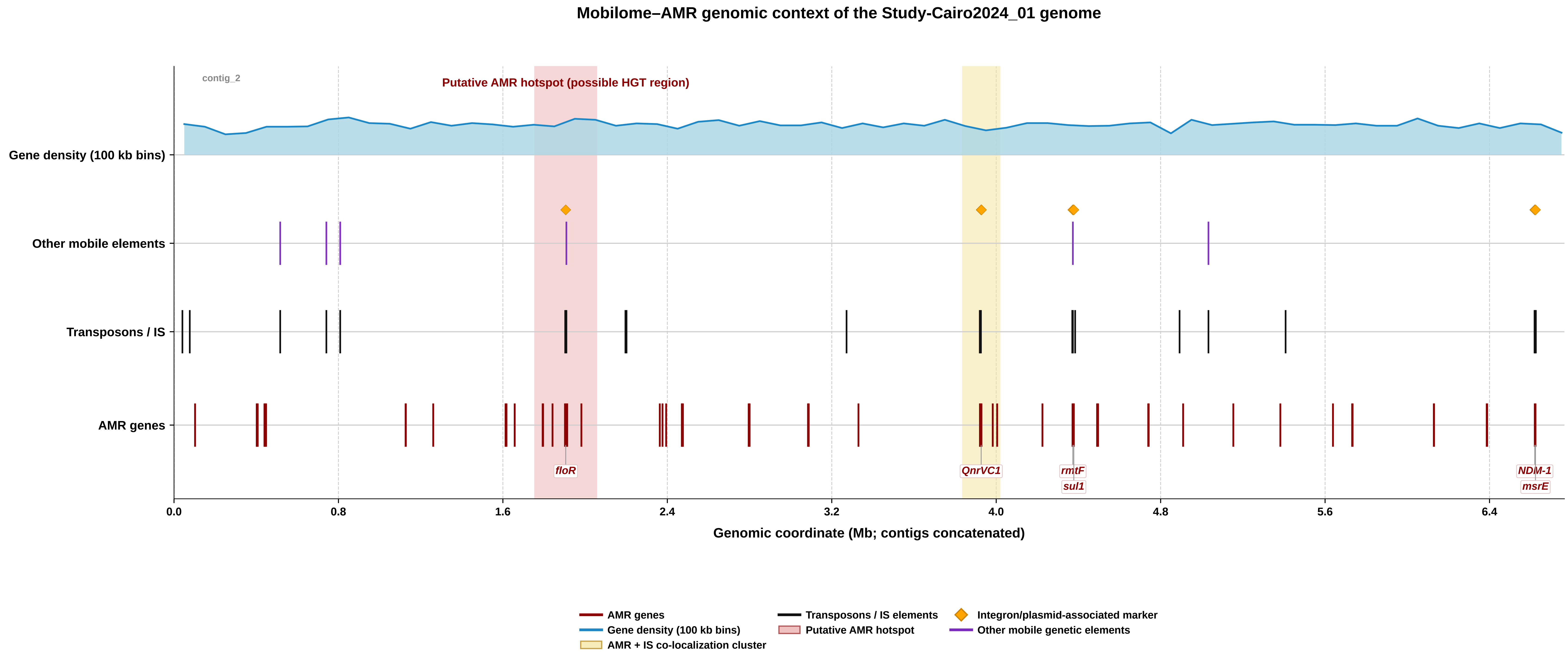

Supplementary Figure S4B

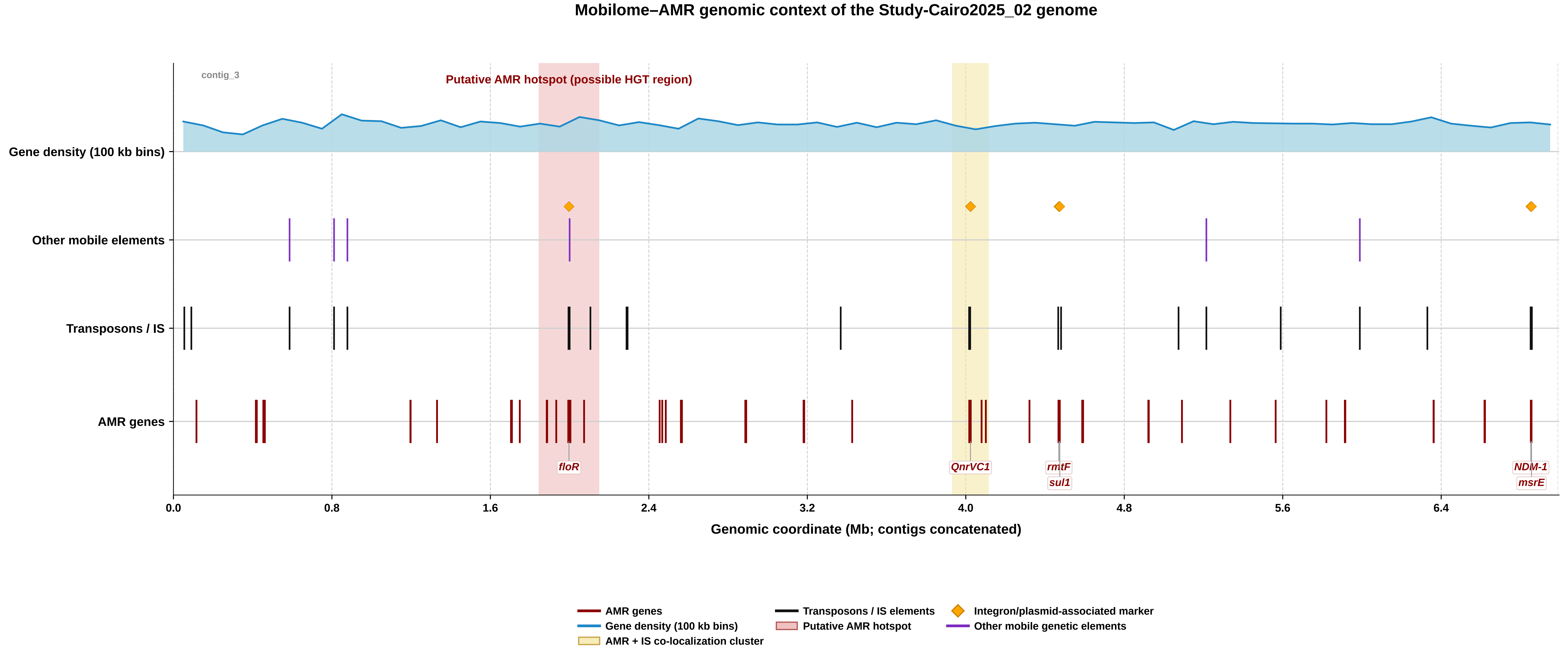

Supplementary Figure S5

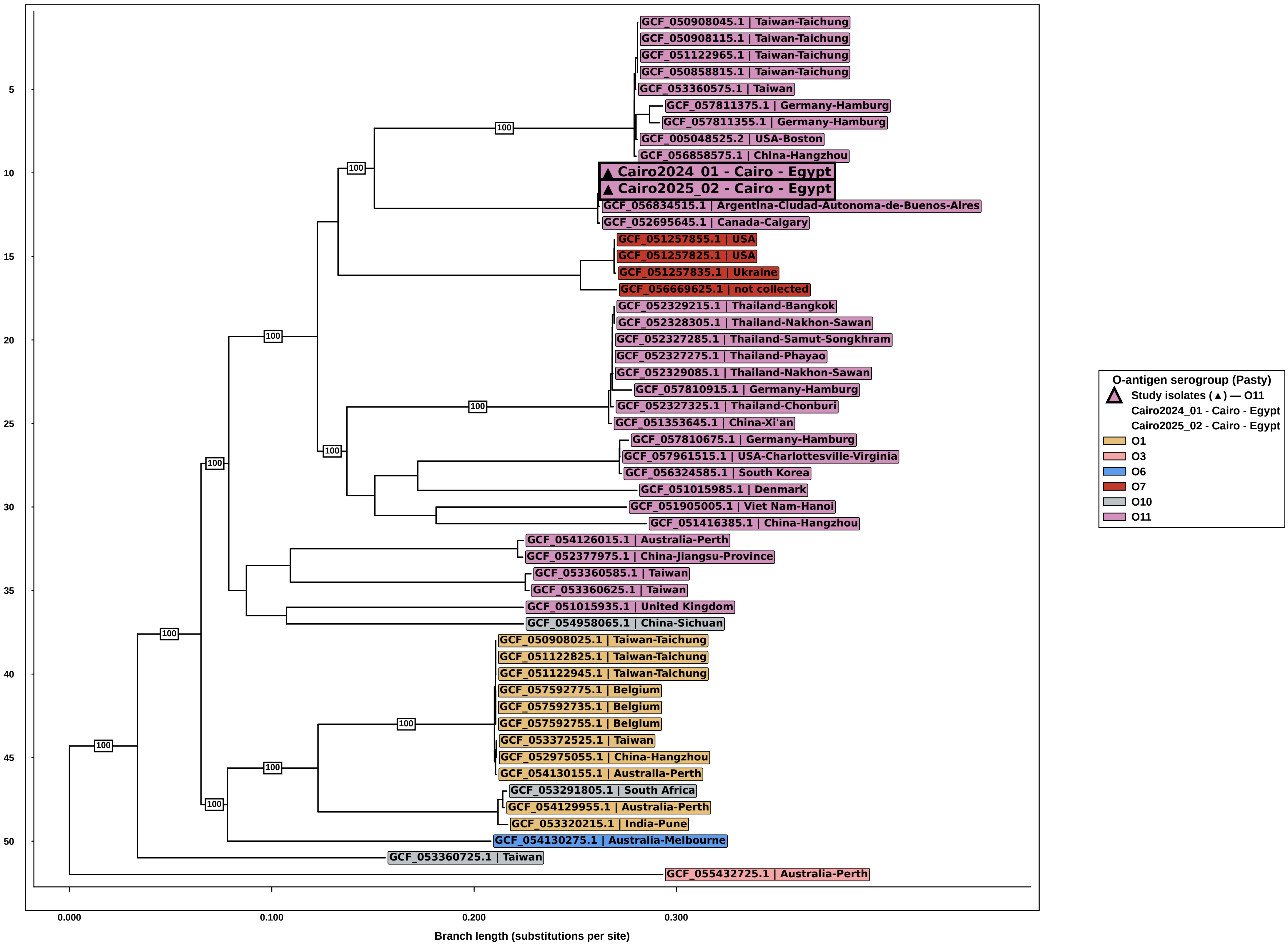

Supplementary Figure S6

Pan-genome presence/absence across 52 P. aeruginosa genomes

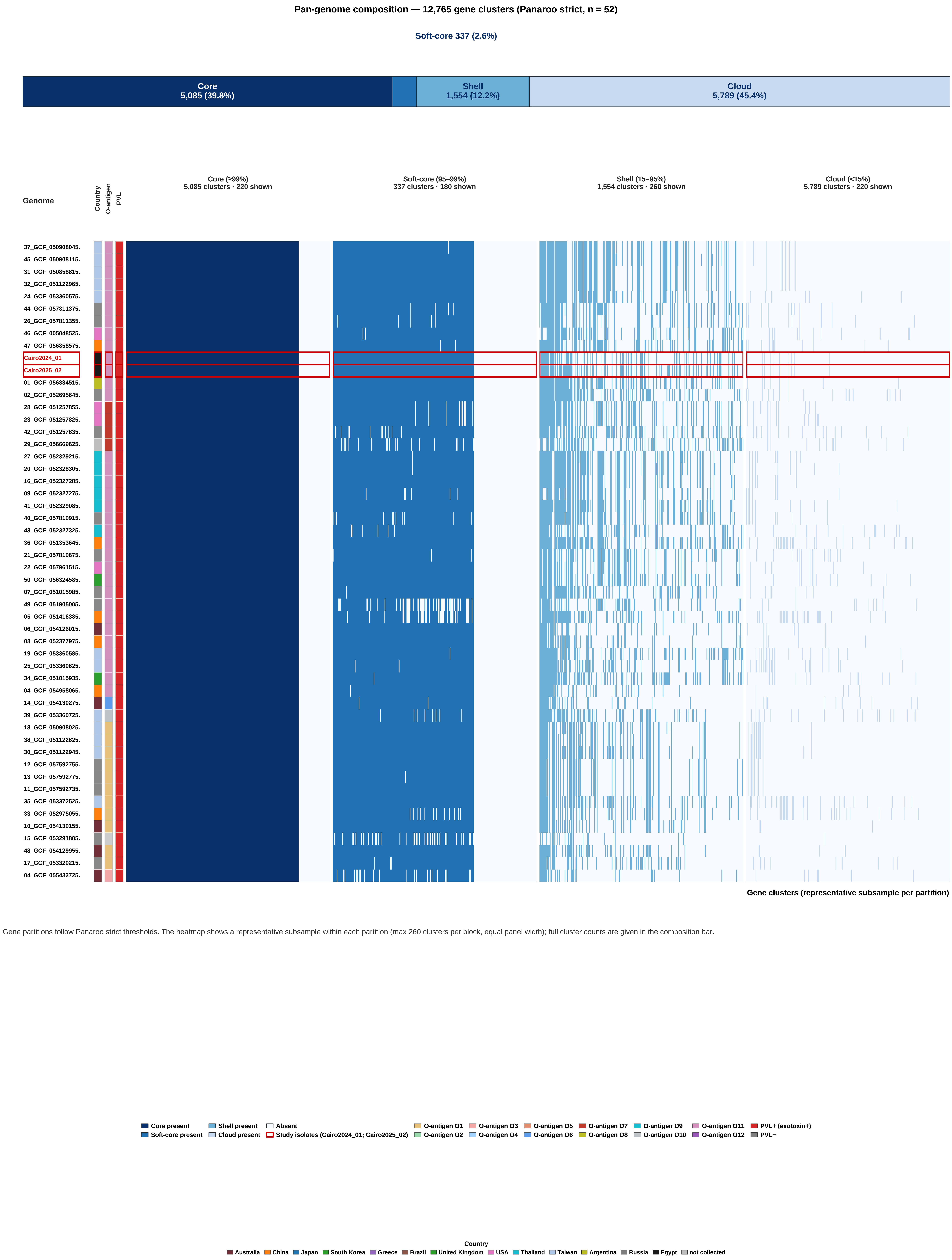

Supplementary Figure S7

AMR, virulence, and IS profiles · 52 *P. aeruginosa* genomes

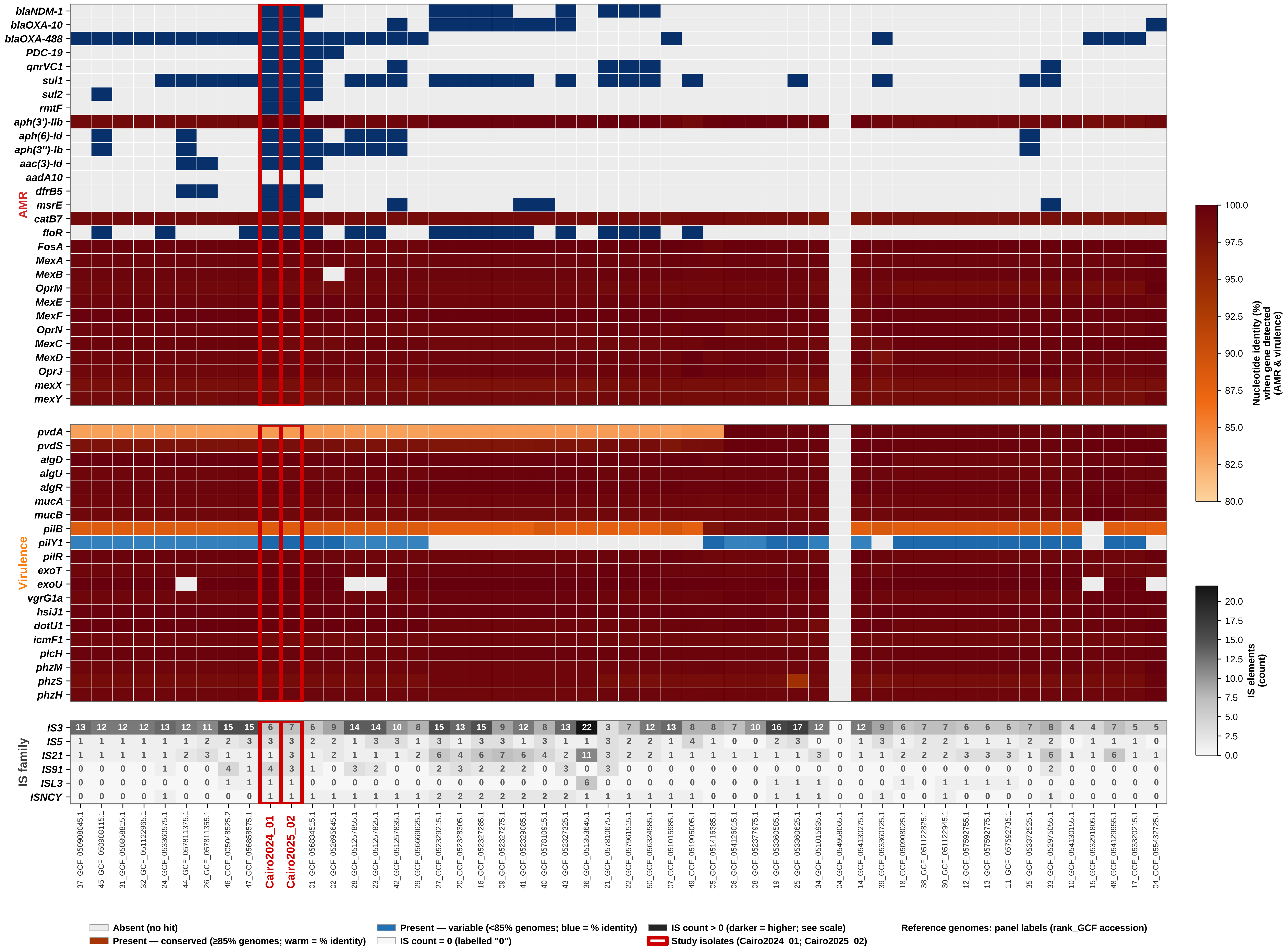

Supplement: Supplementary file 3 [file 12866_2026_5465_MOESM1_ESM.zip › Supplementary_Figures_S1-S7_merged.pdf]
