# Supplementary material for: Comparative genomics of fatal ST308/O11 Pseudomonas aeruginosa from Egypt characterizes conserved resistance architecture, integron-associated multidrug resistance modules, and blaNDM-1 resistance islands
Source: BMC Microbiol. 2026 Aug 8;26:705. doi: 10.1186/s12866-026-05465-y (PMC13452102; doi:10.1186/s12866-026-05465-y)
Supplement: Supplementary file 3 [file 12866_2026_5465_MOESM1_ESM.zip › Figure Legends.docx]

# **Figure Legends:**

**Supplementary Figure S1**

**Supplementary Figure S1.** **Overview of the study design and analytical workflow. A total of 100 clinical samples obtained from ventilator-associated pneumonia (VAP) patients were screened using culture-based identification and antimicrobial susceptibility testing. Two *Pseudomonas aeruginosa* isolates exhibiting the most extensive resistance profiles were selected for whole-genome sequencing. Subsequent analyses included genome assembly and annotation, resistome and virulome profiling, mobile genetic element characterization, multilocus sequence typing (MLST), core-genome SNP phylogenetic reconstruction, pan-genome analysis, and integrated comparative genomic interpretation.**

**Supplementary Figure 2A-B**

**Supplementary Figure S2:** Genome annotation overview and functional subsystem distribution of the two *P. aeruginosa* isolates**.** (A) Circular genome map of Cairo2024_01 (SRR36023598) and (B) Cairo2025_02 (SRR36023597). From outer to inner rings, the maps display genomic coordinates, contigs/chromosomes, coding sequences (CDSs) on the forward strand, CDSs on the reverse strand, non-coding genomic features, antimicrobial resistance (AMR)-associated genes, virulence factor (VF)-associated genes, transporter-associated genes, predicted drug targets, GC content, and GC skew. Functional subsystem distribution of Cairo2024_01 and . Cairo2025_02 based on genome annotation. Functional categories include metabolism, protein processing, stress response/defense/virulence, energy production, membrane transport, cellular processes, DNA processing, RNA processing, cell envelope functions, regulation and cell signaling, and miscellaneous functions. Overall, both isolates exhibited highly similar genome organization and functional annotation profiles, consistent with their close genomic relatedness.

**Supplemntary Figure 3A-B**

**Supplementary Figure S3. Comparative CARD-RGI resistome profiles of the two ST308 Pseudomonas aeruginosa isolates.(A)** Cairo2024_01 (SRR36023598) and **(B)** Cairo2025_02 (SRR36023597). Sunburst plots showing the hierarchical classification of antimicrobial resistance determinants identified using the Comprehensive Antibiotic Resistance Database (CARD) and Resistance Gene Identifier (RGI). The plots demonstrate highly similar resistance gene repertoires between the two isolates, including carbapenemase (blaNDM-1), class D β-lactamases (blaOXA-10 and blaOXA-488), aminoglycoside resistance determinants, fluoroquinolone resistance genes, sulfonamide resistance genes, and multiple efflux-associated components. The predominance of shared resistance determinants is consistent with the closely related genomic backgrounds of the two isolates.

**(Supplementary Figure S4A–B)**

**Supplementary Figure S4: Mobilome–antimicrobial resistance (AMR) genomic context of the two *P. aeruginosa* isolates.** (A) Cairo2024_01 (SRR36023598) and (B) Cairo2025_02 (SRR36023597). Genome-wide distributions of AMR genes (red), insertion sequences and transposon-associated elements (black), and other mobile genetic elements (purple) are shown across the concatenated genome assemblies. Gene density is represented by the blue line (100-kb bins). Shaded regions indicate putative AMR-associated genomic regions characterized by the co-localization of resistance determinants and mobile genetic elements, while orange markers denote integron-associated genomic regions. Selected resistance genes, including blaNDM-1, qnrVC1, sul1, rmtF, floR, and msrE, are annotated to illustrate their genomic context. The overall distribution patterns indicate a largely conserved mobilome architecture between the two isolates. Interpretation of genomic localization should be considered in the context of draft genome assemblies.

**(Supplementary Figure S5)**

**Supplementary Figure S5.** Core-genome SNP-based maximum-likelihood phylogeny of Pseudomonas aeruginosa genomes. The phylogenetic tree was reconstructed from a core-genome SNP alignment comprising 144,617 informative SNP sites identified across 5,085 shared core genes from 52 genomes, including Cairo2024_01 and Cairo2025_02. Maximum-likelihood inference was performed using IQ-TREE under the GTR+ASC substitution model. Branch lengths represent substitutions per site, and the tree was midpoint-rerooted for visualization purposes. Node support values correspond to ultrafast bootstrap and SH-aLRT support where indicated. Egyptian isolates are highlighted and cluster within the ST308 lineage together with publicly available international reference genomes.

**Supplementary Figure S6**

**Supplementary Figure S6.** Pan-genome composition and gene presence–absence patterns across 52 Pseudomonas aeruginosa genomes**.** The pan-genome comprised 12,765 gene clusters identified using Panaroo strict mode, including 5,085 core genes (39.8%), 337 soft-core genes (2.6%), 1,554 shell genes (12.2%), and 5,789 cloud genes (45.4%). The heatmap illustrates representative gene clusters from each partition and their distribution across the 52 analyzed genomes, including the two study isolates (Cairo2024_01 and Cairo2025_02), which are highlighted by red boxes. Colored side annotations indicate country of origin, O-antigen serogroup, and strain metadata. Notably, both study isolates were assigned to the O11 serogroup and clustered within the ST308/O11 lineage represented in the comparative dataset. Dark-colored cells indicate gene presence, whereas light-colored cells indicate gene absence. The predominance of core genes reflects substantial genomic conservation across the dataset, while shell and cloud partitions represent accessory genomic variation among isolates.

**Supplementary Figure S7**

**Supplementary Figure S7. Comparative distribution of antimicrobial resistance genes, virulence-associated genes, and insertion sequence (IS) families across 52 Pseudomonas aeruginosa genomes.** Heatmap showing the presence and nucleotide identity of selected antimicrobial resistance (AMR) genes, virulence-associated genes, and insertion sequence (IS) families among the two study isolates (Cairo2024_01 and Cairo2025_02; highlighted by red boxes) and 50 publicly available reference genomes. For AMR and virulence-associated genes, colored cells indicate gene presence and nucleotide identity relative to reference sequences, whereas white cells indicate gene absence. IS family abundance is represented by numerical counts and grayscale intensity. The two study isolates exhibited highly similar profiles, including the presence of key resistance determinants such as blaNDM-1, blaOXA-10, blaOXA-488, qnrVC1, rmtF, sul1, and multiple efflux-associated components, together with a conserved repertoire of virulence-associated genes and comparable IS family distributions. These findings support the close genomic relatedness of the two ST308 isolates and their highly conserved resistome, virulome, and mobilome characteristics.

**Supplementary Figure S8**

**Supplementary Figure S8. Average nucleotide identity (ANI) comparison of the Egyptian Pseudomonas aeruginosa isolates against selected reference genomes. Pairwise ANI values were calculated between Cairo2024_01 (SRR36023598; blue) and Cairo2025_02 (SRR36023597; red) and a ranked set of reference genomes ordered according to decreasing mean ANI values. The dashed horizontal line indicates the 95% species delineation threshold. The inset panel provides an enlarged view of the high-ANI range to facilitate visualization of minor differences between the two isolates across reference genomes. Both isolates exhibited consistently high ANI values (>99%) throughout the dataset, reflecting their close genomic relationship to the analyzed P. aeruginosa reference genomes.**
